# The 2023 EBMT report on hematopoietic cell transplantation and cellular therapies. Increased use of allogeneic HCT for myeloid malignancies and of CAR-T at the expense of autologous HCT

**DOI:** 10.1038/s41409-025-02524-2

**Published:** 2025-02-12

**Authors:** Jakob R. Passweg, Helen Baldomero, Marina Atlija, Iliana Kleovoulou, Aleksandra Witaszek, Tobias Alexander, Emanuele Angelucci, Dina Averbuch, Ali Bazarbachi, Fabio Ciceri, Raffaella Greco, Mette D. Hazenberg, Krzysztof Kalwak, Donal P. McLornan, Bénédicte Neven, Zinaida Perić, Antonio M. Risitano, Annalisa Ruggeri, Isabel Sánchez-Ortega, John A. Snowden, Anna Sureda

**Affiliations:** 1 EBMT Activity Survey Office Basel, Hematology Division, University Hospital, Basel, Switzerland; 2 EBMT Activity Survey Office Leiden, Leiden, The Netherlands; 3 Department of Rheumatology and Clinical Immunology, Charité—Universitätsmedizin Berlin, Berlin, Germany; 4 Hematology and Cellular Therapy Unit, IRCCS Ospedale Policlinico San Martino, Genova, Italy; 5 Faculty of Medicine, Hadassah Medical Center, Hebrew University of Jerusalem, Jerusalem, Israel; 6 Bone Marrow Transplantation Program, Department of Internal Medicine, American University of Beirut, Beirut, Lebanon; 7 Unit of Hematology and Bone Marrow Transplantation, IRCCS San Raffaele Hospital, Vita-Salute San Raffaele University, Milan, Italy; 8 Department of Hematology, Amsterdam University Medical Centres, University of Amsterdam, Amsterdam, The Netherlands; 9 Department of Hematopoiesis, Sanquin Research, Amsterdam, The Netherlands; 10 Clinical Department of Pediatric BMT, Hematology and Oncology, Wroclaw Medical University, Wroclaw, Poland; 11 Department of Haematology, University College London Hospitals NHS Foundation Trust, London, UK; 12 Pediatric Immune-Hematology Unit, Necker Children Hospital, Assistance Publique Hôpitaux de Paris, Paris, France; 13 University Hospital Centre Rijeka, Rijeka, Croatia; 14 Hematology and Hematopoietic Transplant Unit, Azienda Ospedaliera di Rilievo Nazionale San Giuseppe Moscati” (A.O.R.N. Giuseppe Moscati), Avellino, Italy; 15 EBMT Executive Office, Barcelona, Spain; 16 Department of Haematology, Sheffield Teaching Hospitals NHS Foundation Trust, Sheffield, UK; 17 Clinical Hematology Department, Institut Català d’Oncologia-Hospitalet, Institut d’Investigació Biomèdica de Bellvitge (IDIBELL), University of Barcelona, Barcelona, Spain

**Keywords:** Leukaemia, Lymphoma

## Abstract

In 2023, 47,731 HCT (20,485 (42.9%) allogeneic and 27,246 (57.1%) autologous) in 43,902 patients were reported by 696 European centers. 6042 patients received advanced cellular therapies, 4888 of which were CAR-T. Compared to the previous year there was an increase in CAR-T (+52.5%), in allogeneic HCT (+7.8%) but none in autologous HCT (+0.4%). Main indications for allogeneic HCT were myeloid (11,748; 60.7%), lymphoid malignancies (4,850; 25.0%), and non-malignant disorders (2558; 13.2%). Use of allogeneic HCT increased for AML (+12.1%) and for NHL (+11.0%), particularly in T-NHL (+25.6%). Main indications for autologous HCT were lymphomas (7890; 32.2%), PCD (14,271; 58.2%), and solid tumors (1608; 6.6%) with recovering numbers for autoimmune diseases. In patients with allogeneic HCT, the use of sibling donors increased by +1.0%, haploidentical donors by +11.7%, and unrelated donors by +11.1%. Cord blood HCT decreased again by −5.4%. Pediatric HCT activity increased slightly (5455; +0.1%) with differences between allogeneic (4111; −0.5%) and autologous HCT (1344: +1.7%). Use of CAR-T increased to a cumulative total of 13,927 patients including patients treated for autoimmune diseases. Overall, numbers show a complete recovery from the pandemic dip with increased cellular therapy at the expense of autologous HCT. Allogeneic HCT activity focuses on myeloid malignancies.

## Introduction

The European Society for Blood and Marrow Transplantation (EBMT) has published a survey since 1990 [1] describing activity in hematopoietic cell transplantation (HCT) centers in Europe, updated annually thereafter. The survey, now spanning 34 years, includes patients receiving more than 990,800 transplants approaching the one million transplants landmark. The survey was originally designed in the form of a single-page spreadsheet for ease of reporting and has remained in this format until 2022. For the 2023 annual survey, an online data reporting system was developed, enabling centers to submit their data electronically and update any changes that occurred within the center over the past year. Many additional features have been added over the years, such as extended disease classification, donor type and stem cell source, information on conditioning intensity and pediatric activity, the addition of non-HCT treatments, such as cellular therapies and more recently immunosuppressive treatment (IST) for marrow failure.

HCT is an established procedure for many acquired or inherited disorders of the hematopoietic system, benign or neoplastic, including those of the immune system, and to facilitate enzyme replacement in metabolic disorders [2–4]. The activity survey of the EBMT, describing the status of HCT, has become an instrument to observe trends and monitor changes in HCT technology in Europe and neighboring countries [5–15]. The survey, using a standardized structure, captures the number of HCTs from highly committed participating centers, stratified by indication, donor type, and stem cell source over time [16–19]. In more recent years, the survey also included information on cellular therapies, qualifying as medicinal products with hematopoietic cells for uses other than to replace the hematopoietic system [19, 20]. The analysis of the survey data since 1990 has illustrated a continued and constant increase in the annual number of HCT and transplant rates for both allogeneic and autologous HCT. This continued rise came to an abrupt stop in 2020, likely driven by the SARS-CoV-2 pandemic [14]. This 2023 survey data show that after the limited recovery observed in the second year of the pandemic, the overall trend of continuous growth seen in allogeneic HCT in earlier years appears to once again be evident. This is, however, less so for autologous HCT.

## Patients and methods

### Data collection and validation

For the first time in 2023, centers were asked to report their data directly via an online data entry system. In addition to submitting detailed data within the activity survey tables, the center also had the possibility to update any changes that occurred within the center, i.e., change of staff. The main part of the activity survey remained the same as in previous years where patients receiving their first transplant in the survey year were reported by disease, donor type, and stem cell source in the main table. Additional information on the number of subsequent transplants performed due to relapse, rejection, or those that are part of a planned sequential protocol is reported in summative form (Table 1). Information on the number of pediatric HCTs, those patients receiving un-manipulated donor lymphocyte infusion (DLI), and non-myeloablative or reduced-intensity HCT were also collected. New to the 2023 survey was the possibility to report the number of patients receiving IST for bone marrow failure (BMF).

**Table 1:** Number of patients receiving a first allogeneic or first autologous HCT in 2023 by indication, donor type, and stem cell source.

	Transplant activity 2023																		
	No. of patients																		
	Allogeneic													Autologous			T o t a l		
	Family										Unrelated						Allo	Auto	Total
	HLA-id			Twin		Haplo ≥ 2 MM		Other family						BM	BM +				
	BM	PBSC	Cord	BM	PBSC	BM	PBSC	BM	PBSC	Cord	BM	PBSC	Cord	only	PBSC	Cord			
Myeloid malignancies	214	2283	2	2	21	248	1738	3	71	1	308	6748	109	0	191	1	11,748	192	11,940
Acute myeloid leukemia	152	1626	2	1	19	181	1259	2	44	1	177	4250	88	0	178	1	7802	179	7981
1st complete remission	94	1055	1	1	19	97	691	1	28	1	96	2393	45	0	158	0	4522	158	4680
Not 1st complete remission	45	336	0	0	0	61	356	1	10	0	56	955	29	0	18	1	1849	19	1868
AML therapy related	9	73	0	0	0	5	65	0	1	0	11	239	5	0	1	0	408	1	409
AML with MDS-related changes	4	162	1	0	0	18	147	0	5	0	14	663	9	0	1	0	1023	1	1024
Chronic myeloid leukemia	6	88	0	0	0	8	44	0	5	0	17	193	5	0	5	0	366	5	371
Chronic phase	3	46	0	0	0	4	18	0	2	0	10	93	4	0	5	0	180	5	185
Not chronic phase	3	42	0	0	0	4	26	0	3	0	7	100	1	0	0	0	186	0	186
MDS or MDS/MPN overlap	51	379	0	1	2	53	319	1	18	0	104	1629	16	0	7	0	2573	7	2580
MPN	5	190	0	0	0	6	116	0	4	0	10	676	0	0	1	0	1007	1	1008
Lymphoid malignancies	269	1111	0	1	11	194	859	7	47	0	226	2061	64	5	22,200	0	4850	22,205	27,055
Acute lymphatic leukemia	238	711	0	1	7	149	520	3	22	0	202	1182	54	0	39	0	3089	39	3128
1st complete remission	131	501	0	0	6	81	267	0	14	0	106	799	25	0	36	0	1930	36	1966
Not 1st complete remission	107	210	0	1	1	68	253	3	8	0	96	383	29	0	3	0	1159	3	1162
Chronic lymphocytic leukemia	0	35	0	0	0	1	14	1	1	0	0	122	0	0	5	0	174	5	179
Plasma cell disorders—MM	1	33	0	0	2	2	12	0	9	0	0	99	0	1	13802	0	158	13803	13961
Plasma cell disorders—other	3	5	0	0	1	1	2	0	0	0	0	17	1	1	467	0	30	468	498
Hodgkin lymphoma	6	87	0	0	0	14	104	2	4	0	3	143	3	1	2280	0	366	2281	2647
DLBCL NHL all types	2	77	0	0	0	7	57	0	6	0	3	126	1	2	2836	0	279	2838	3117
Other B-cell NHL	2	39	0	0	1	6	34	0	1	0	3	75	0	0	1988	0	161	1988	2149
T cell NHL	17	124	0	0	0	14	116	1	4	0	15	297	5	0	783	0	593	783	1376
Solid tumors	1	3	0	0	0	2	25	0	1	0	0	6	0	5	1603	0	38	1608	1646
Neuroblastoma	1	1	0	0	0	2	23	0	0	0	0	1	0	1	570	0	28	571	599
Soft tissue sarcoma	0	2	0	0	0	0	0	0	0	0	0	1	0	2	58	0	3	60	63
Ewing sarcoma	0	0	0	0	0	0	1	0	0	0	0	0	0	1	161	0	1	162	163
Germinal tumors	0	0	0	0	0	0	0	0	1	0	0	0	0	1	480	0	1	481	482
Other solid tumors	0	0	0	0	0	0	1	0	0	0	0	4	0	0	334	0	5	334	339
Non-malignant disorders	638	360	17	4	4	197	200	75	56	0	447	527	33	4	512	1	2558	517	3075
Bone marrow failure—SAA	165	135	0	3	4	59	49	8	6	0	150	161	2	1	1	0	742	2	744
Bone marrow failure—other	71	36	1	0	0	15	17	12	7	0	60	46	3	0	0	0	268	0	268
Thalassemia	98	49	8	1	0	10	8	15	12	0	44	66	0	0	4	0	311	4	315
Sickle cell disease	162	91	4	0	0	53	28	20	8	0	15	11	1	1	2	0	393	3	396
Inborn errors of immunity	106	37	4	0	0	56	80	18	14	0	126	172	11	2	0	0	624	2	626
Inborn errors of metabolism	31	8	0	0	0	4	16	2	9	0	50	59	15	0	1	1	194	2	196
Autoimmune disease—MS	1	2	0	0	0	0	0	0	0	0	0	5	0	0	419	0	8	419	427
Autoimmune disease -SSC	0	0	0	0	0	0	0	0	0	0	0	0	0	0	51	0	0	51	51
Autoimmune disease—other	4	2	0	0	0	0	2	0	0	0	2	7	1	0	34	0	18	34	52
Others	25	29	1	0	0	16	19	5	3	0	26	47	3	0	12	0	174	12	186
Total patients	1147	3786	20	7	36	657	2841	90	178	1	1007	9389	209	14	24,518	2	19,368	24,534	43,902
Re/additional transplants	28	118	0	0	1	48	328	6	9	0	45	508	26	3	2709	0	1117	2712	3829
Total transplants	1175	3904	20	7	37	705	3169	96	187	1	1052	9897	235	17	27,227	2	20,485	27,246	47,731
N. Pediatric patients (<18 at HCT)	857	388	17	5	4	351	565	83	66	0	729	917	129	12	1330	2	4111	1344	5455

In addition, in Table 2, centers reported information on different types of cellular therapies qualifying as advanced therapy medicinal products (ATMPs). These therapies result from substantial manipulations of collected cells, whether manufactured centrally by industry or locally by an academic institution.

**Table 2:** Number of patients treated with non-HCT cellular therapies in 2023 by indication, donor type, and cell source.

Number of patients	*DLI*	CAR-T		MSC		NK cells		Selected/Expanded T cells or CIK		Regulatory T cells (TREGS)		Genetically modified T cells		Dendritic cells		Expanded CD34+ cells		Genetically modified CD34+ cells		Other		Total excluding DLI	
		Allo	Auto	Allo	Auto	Allo	Auto	Allo	Auto	Allo	Auto	Allo	Auto	Allo	Auto	Allo	Auto	Allo	Auto	Allo	Auto	allo	auto
GvHD				326	5			3		25						1	1			11	5	366	11
Graft enhancement				46				4		1	1					8		3		144	46	206	47
Autoimmune disease			36	31	4																3	31	43
Genetic disease								4											12	5		9	12
Infection				1		3		108	7	2		16						1		10		141	7
Malignancy—ALL		47	519		2	15		11	4					3		1				9		86	525
Malignancy—HL/NHL		25	3437			12		20	1		5									1	11	58	3454
Malignancy—Myeloma		0	736			25	2														11	25	749
Malignancy - other indication		12	76	11	8	8	1	14	8		5		14	13		2			4	42	54	102	170
*DLI for graft enhancement/failure*	*686*																						
*DLI for residual disease*	*432*																						
*DLI for relapse*	*1299*																						
*DLI per protocol*	*458*																						
Total	*2875*	84	4804	415	19	63	3	164	20	28	11	16	14	16	0	12	1	4	16	222	130	1024	5018

Quality control measures included several independent systems: confirmation of the validity of data entered by the center directly after data submission via an output of the data entered, and cross-checking with selected National Registries.

### Participating centers

Since 1990, a directory of HCT and cellular therapy centers consisting of both members of the EBMT and non-members, in both European and collaborating non-European countries has been accrued. The directory is updated annually according to the center’s current activity. In 2023, 741 centers from 54 countries were contacted (44 European and 10 collaborating countries) of which 696 centers responded. This corresponds to a 93.9% return rate and includes 16.1% EBMT non-members. Forty-five active centers failed to report in 2023. When compared with previously reported data from these centers, it accounts for ~2000 non-reported HCTs.

Participating centers are listed in the Supplementary Online Appendix in alphabetical order, by country, city, and EBMT center code, with the reported number of first and total HCT, and of first allogeneic and autologous HCT. The WHO regional office definitions were used to classify countries as European or non-European. Ten collaborating non-European countries participated in the 2023 survey: Algeria, Iran, Iraq, Jordan, Lebanon, Nigeria, Saudi Arabia, South Africa, Tunisia, and the United Arab Emirates. Their data, 3025 HCT in 2894 patients, from 30 active transplant centers made up 6.4% of the total dataset and are included in all analyses.

### Patient and transplant numbers

Wherever appropriate, patient numbers corresponding to the number of patients receiving a first transplant in 2023, and transplant numbers reflecting the total number of transplants performed were listed. The term sibling donor included HLA-identical siblings and twins but not siblings with HLA mismatches. Haploidentical transplants refer to those from any family member with at least a full haplotype mismatch. Other family member donors were those related donors that are mismatched to a lesser degree than a full haplotype. For the analysis, we added the small number of “other family donors” to haploidentical donor HCT. Unrelated donor transplants included HCT from matched or mismatched unrelated donors. Stem cell source included cells collected from bone marrow (BM), peripheral blood (PBSC), or cord blood (CB). Additional non-first transplants included multiple transplants defined as subsequent transplants within a planned double or triple autologous or allogeneic transplant protocol. Re-transplants (autologous or allogeneic) were defined as unplanned HCT for either rejection, poor-graft function, or relapse after a previous HCT. Further information includes the number of myeloablative or non-myeloablative HCT and patients receiving DLI post-transplant.

### Hematopoietic advanced cellular therapies other than hematopoietic cell transplantation

Centers reported patients receiving cellular therapies other than HCT. Hematopoietic advanced cellular therapies were defined as infusion of cells undergoing substantial manipulation after collection, either selection and/or expansion, or genetic modification and thus qualify as investigational or ATMPs. Depending on their nature and indications, hematopoietic cellular therapies may be designed to replace or to complement HCT. Administration of non-substantially manipulated hematopoietic cells, such as transplantation of CD34+ selected hematopoietic stem cells were counted as HCT and not as cellular therapy [18]. Similarly, un-manipulated lymphocyte infusions post-HCT were counted as DLI and not as ATMPs. Hematopoietic cellular therapies include immune effector cells as defined in FACT-JACIE standards for Hematopoietic Cellular Therapy. This definition covers chimeric antigen receptor T cells (CAR-T) cells and forms the basis for accreditation requirements in recent EBMT-JACIE recommendations [17, 19, 20].

Hematopoietic cellular therapies were categorized as CAR-T, in vitro selected/ and or expanded T cells or cytokine activated, such as virus-specific T cells; cytokine-induced killer cells (CIK); regulatory T cells (TREGS); genetically modified T cells other than CAR-T; natural killer cells (NK); dendritic cells; mesenchymal stromal cells; in vitro expanded CD34+ cells; and genetically modified CD34+ cells. This survey did not include cells from sources other than hematopoietic tissue.

### Transplant and cellular therapy rates

Transplant rates, defined as the total number of HCT per 10 million inhabitants (10^7^), were computed for each country, without adjusting for patients receiving their HCT in a foreign country or for refugee populations not considered inhabitants. Cellular therapy rates were defined as the number of patients receiving a CAR-T cellular therapy treatment per 10 million inhabitants. Population numbers for the European countries in 2023 were obtained from Eurostats: (https://ec.europa.eu/eurostat) and the World Bank database for the non-European countries: (https://databank.worldbank.org).

### Analysis

Wherever appropriate, the absolute number of transplanted patients, number of transplants, or transplant rates are shown for specific countries, indications, or transplant techniques. Myeloid malignancy includes acute myeloid leukemia (AML), myelodysplastic neoplasm or myelodysplastic/myeloproliferative overlap syndrome (MDS or MDS/MPN overlap syndrome), myeloproliferative neoplasm (MPN), and chronic myeloid leukemia (CML). Lymphoid malignancy includes acute lymphocytic leukemia (ALL), chronic lymphocytic leukemia (CLL), Hodgkin lymphoma (HL), non-Hodgkin lymphoma (NHL), and plasma cell disorders (PCD) (including multiple myeloma (MM) and other PCD). Non-malignant disorders include BMF (severe aplastic anemia (SAA) and other BMF), thalassemia and sickle cell disease (HG), inborn errors of immunity (IEI), inborn errors of metabolism (IEM), and autoimmune diseases (AID). Others include histiocytosis and other rare disorders.

## Results

### Participating centers in 2023

Of the 696 responding centers, 451 (64.8%) performed both allogeneic and autologous transplants; 219 (31.5%) restricted their activity to autologous HCT only and 21 (3.0%) to allogeneic HCT only. Five of the 696 responding centers (0.7%; 2 adult and 3 pediatric centers) reported no activity due to renovation or changes within the transplant unit. Of the 691 actively transplanting centers in 2023, 454 (65.7%) performed transplants for adults only, and 116 (16.8%) performed transplants for both adult and pediatric patients. An additional 121 (17.5%) were dedicated pediatric-only transplant centers.

### Number of patients, transplants, and trends in 2023

**Fig. 1:**
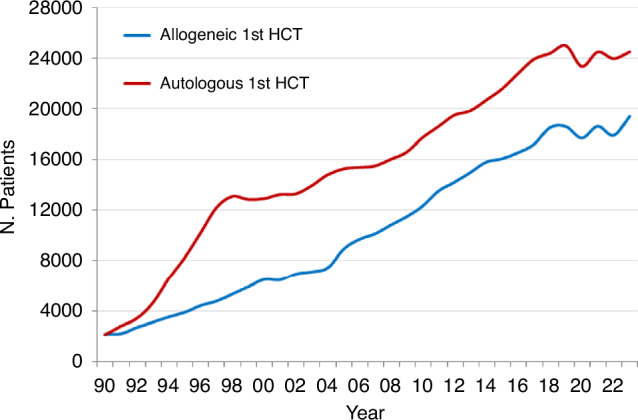
Number of patients receiving the first allogeneic or autologous HCT from 1990 to 2023.

In 2023, 47,731 transplants were reported in 43,902 patients; of these, 20,485 HCT (42.9%) were allogeneic and 27,246 (57.1%) autologous (Table 1, Fig. 1). When compared to the 2022 data, an overall increase of +3.4% was observed (+7.8% allogeneic HCT and +0.4% autologous HCT). In addition, there were 3829 second or subsequent transplants. Of these,1117 were allogeneic HCT, given mainly to treat relapse or graft failure and 2712 were autologous, the majority of which were part of multiple transplant procedures such as tandem HCT, to treat relapse, or as salvage autologous transplants for PCD. A further 493 patients receiving their first allogeneic HCT were reported as being given after a previous autologous HCT. The main indications being lymphoproliferative disorders such as NHL (*n* = 145), HL (*n* = 120), or PCD (*n* = 85) and AML (*n* = 61).

### Main indications

**Fig. 2:**
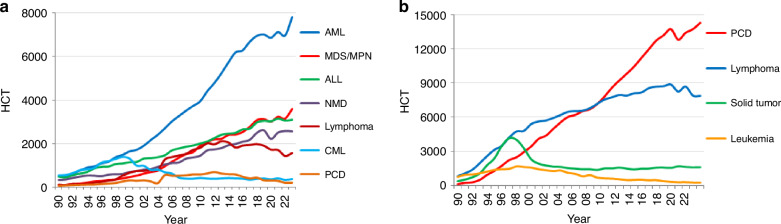
Change in main disease indication for allogeneic and autologous HCT 1990 to 2023. **a** Allogeneic HCT. **b** Autologous HCT.

Indications for HCT in 2023 are listed in detail in Table 1. Main indications for patients receiving an allogeneic HCT were myeloid malignancies; 11,748 (AML, CML, MDS or MDS/MPN overlap syndrome and MPN) (Fig. 2a). For autologous HCT, the main indications were lymphoid malignancies; 22,205 (ALL, CLL, PCD, HL and NHL) (Fig. 2b). The distribution of disease indication for allogeneic HCT (Supplementary Fig. 1a) and autologous HCT (Supplementary Fig. 1b) is shown as pie graphs color coded by disease group.

### Changes observed in allogeneic HCT from 2022 to 2023

During the years 2020–2022, many transplant centers were affected by the SARS-CoV-2 pandemic and fluctuations were observed in HCT activity in a wide range of disease indications throughout numerous countries in Europe and worldwide. However, this year’s 2023 activity report shows again an overall increase in many indications with certain notable exceptions marking the end of the pandemic years. Compared to the 2022 activity report, an overall increase in allogeneic HCT of +7.8% is observed vs the decrease of −4.0% reported in 2022 (Fig. 1). Within the leukemias (AML, ALL, MDS, or MDS/MPN overlap syndrome, CML and CLL; *n* = 15,011), the overall decrease of −3.1% reported between the years 2021 and 2022 increased by +10.0% in 2023. AML (all stages), the leading indication for allogeneic HCT accounting for 40% of all allogeneic HCT increased overall by +12.1% (Fig. 2a). Early-stage disease AML increased by +8.2%, therapy-related AML or AML with myelodysplasia related changes by +25.5% and advanced stage AML by +12.7%. Allogeneic HCT for CML increased by +9.8% for early stage and by +11.4% for late-stage disease. For MDS an increase of +12.8% and for MPN of +17.1% was observed. HCT for ALL, which comprises of 16% of all allogeneic HCT, increased primarily for early-stage disease (+2.4%), with a slight decrease of −0.7% for late-stage disease. CLL increased again by +10.8% after the drop seen between the years 2021 and 2022 of −16.9% and for NHL, an increase of +11.0% was observed (−16.5% in 2022). In addition, enhanced definition within NHL was introduced in the 2022 survey, enabling the possibility to look at the emerging trends taking place within this particular indication. Allogeneic transplant numbers increased for HL (+5.2%) and NHL overall (+11.0%), particularly for DLBCL (279 (+5.7%); 264 in 2022) and T cell NHL (593 (+25.6%); 472 in 2022). However, for other B-cell NHL, a decrease was observed (161 (−17.4%); 195 in 2022). Within the non-malignant disorders, an overall decrease of −0.5% was reported. BMF-SAA activity decreased by −5.7% (+9.3% in 2022) and BMF non-SAA by −3.6% (+4.5% in 2022). Overall, 742 patients received an allogeneic HCT for SAA in 2023, compared to the reported 605 patients treated with immunosuppression (IST). Within the hemoglobinopathies, the number of allogeneic HCT for thalassemia decreased by −12.6% after an increase of +23.2% reported in 2022. Allogeneic HCT for sickle cell disease however continued to rise, +18.0% (+2.8% in 2022). Reported activities for IEI and IEM remained similar to numbers reported in recent years, with a slight decrease of −1.9% for IEI (*n* = 624, 636 in 2022) and an increase of +26.8% observed for IEM (*n* = 194, 153 in 2022). Within the total numbers of allogeneic HCT, 9092 (44.4%) were performed using non-myeloablative or reduced-intensity conditioning in 2023. This is an increase of +17.0% when compared to 2022 (*n* = 7772).

### Allogeneic HCT for myeloid malignancies

**Fig. 3:**
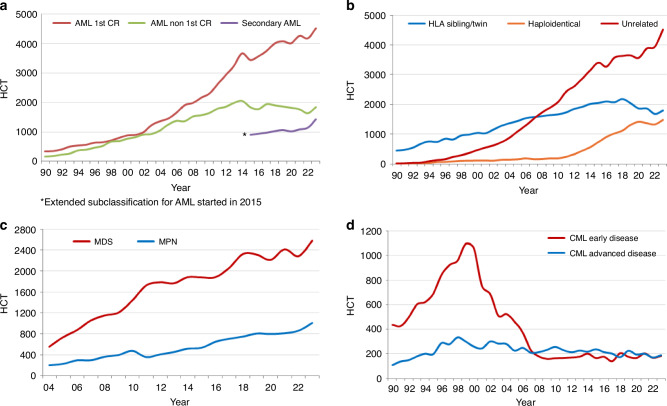
Major trends in disease indication and type of donor for allogeneic HCT from 1990 to 2023. **a** Allogeneic HCT for AML in early stage, late stage, and secondary AML from 1990 to 2023. **b** Change in type of donor in AML (all stages) from 1990 to 2023. **c** Allogeneic HCT for MDS or MDS/MPN overlap and MPN from 2004 to 2023. **d** Allogeneic HCT for CML in early and late stages from 1990 to 2023.

Figure 3a–d shows trends in allogeneic HCT for myeloid malignancies. Allogeneic HCT for AML continues to increase in patients transplanted in CR1. HCT for advanced disease increased by +12.7% in 2023 but the overall trend since 2014 indicates annual fluctuation rather than the steady increase observed in earlier years. Patients transplanted with secondary or post cytotoxic chemotherapy AML increased by +25.5% (+5.4% in 2022) (Fig. 3a). We observed a major increase in the use of unrelated donors while the use of sibling donors and haploidentical donors remained more or less stable since the pandemic (Fig. 3b). In Fig. 3c allogeneic HCT for MDS and MPN is shown with a major increase in HCT in 2023 for MDS (+12.8%) compared to the fluctuation observed between the years 2019 to 2022 and an ongoing increase for MPN (+17.1%, +6.0% in 2022). Figure 3d shows allogeneic HCT for CML with a remarkable stability (*n* = 400) in patients receiving allogeneic HCT in the chronic phase i.e., TKI refractory, or in the more advanced disease phase since 2008, possibly indicating that access to TKI is now universal in the countries monitored and the absence of changes in indications for CML HCT.

### Changes in donor type and stem cell source from 2022 to 2023

**Fig. 4:**
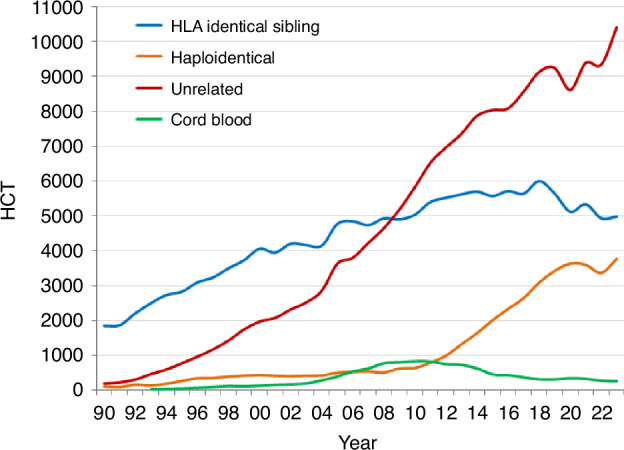
Change in type of donor for first allogeneic HCT from 1990 to 2023.

In 2023, an increase was reported in HCT in all donor types. The overall number of all transplants for each donor type including BM, peripheral blood stem cells, or CB as stem cell source was 5143 for HLA-identical sibling donor, 4158 for haploidentical and other family relative donor and 11,184 (54.6%) for unrelated donor. Looking at the trends over time for patients receiving an allogeneic HCT and treated with an HLA-identical sibling, a slight increase of +1.0% (−7.7% in 2022) was observed. With haploidentical donors, where a drop had been observed in 2020-2021, an increase of +11.7% (−6.3% in 2022) was once again seen. For unrelated donors, an increase was seen of +11.1% when compared to the decrease of −1.0% reported in 2022. (Fig. 4). The overall use of CB HCT continued to decrease by −6.2%, an ongoing trend observed in recent years, after a small increase in 2020 most likely related to the SARS-CoV-2 pandemic. In patients receiving an allogeneic HCT, the stem cell source of choice was PBSC, 83.9% (*n* = 17,194) for BM, 14.8% (*n* = 3035), and for CB transplants 1.3% (*n* = 256). The choice of stem cell source varied according to the patient’s disease and age, with higher numbers of PBSC being used in diseases of adulthood when compared to pediatric disease.

### Changes in autologous HCT from 2022 to 2023

As seen in allogeneic HCT, fluctuations were also observed over the 3 years of the pandemic for autologous HCT. The numbers observed from 2020 to 2023 seemed to stabilize with a slight overall increase of +0.4% when compared to 2022 (Fig. 1b). The highest number of autologous HCT ever reported was in 2019 (for allogeneic HCT the number reported in 2023 was slightly higher than the pre-pandemic high).

The main indications for autologous HCT were lymphoid malignancies (*n* = 22,205), with PCD comprising 58.2% of all autologous HCT indications (*n* = 14,271). Autologous HCT activities for PCD increased +4.2% (+2.4% in 2022) and HL by +2.8% (−3.4% in 2022) but declined for NHL overall by −1.2% (−10.5% in 2022). Of the autologous transplants for NHL (*n* = 5609, 5679 in 2022), the majority were for DLBCL (*n* = 2838; 50.6% of NHL), other B-cell NHL (*n* = 1988; 35.4%) and a smaller number for T cell NHL (*n* = 783; 14.0%) as the reported indication. In solid tumors, autologous HCT numbers increased slightly from 1593 in 2022 to 1608 in 2023 (+0.9%) and have remained stable for the last 10 years. For all types of AML, the decrease in autologous HCT activity has continued in 2023 by −13.1% (−2.8% in 2022). For AID (*n* = 504), the overall decrease of −44.7% (*n* = 298) seen in 2020 is now gradually recovering to numbers similar to those prior to the pandemic (*n* = 539). The main indication for autologous HCT in AID is multiple sclerosis (*n* = 419), followed by systemic sclerosis (*n* = 51) and 34 in patients with other types of AID.

### Pediatric transplantation in 2023

The number of pediatric patients (<18 years old at transplant) transplanted in both dedicated pediatric and joint adult-pediatric units was 5455 (4111 allogeneic; 75.4% and 1344 autologous) (Table 1). This is an overall increase of +0.1% in the total number of transplants, with a decrease of −0.5% in allogeneic HCT but an increase of +1.7% in autologous HCT when compared to 2022. Among allogeneic HCT, BM stem cells were used in 2025 patients (49.4%), of which 729 (36.0%) were from unrelated donors and peripheral blood stem cells in 1929 patients (47.1%), of which 914 (47.7%) were from unrelated donors. CB stem cells were used in 146 (3.6%) pediatric patients which accounts for 63.5% of all patients receiving a CB transplant in 2023 (*n* = 230). Of the 146 pediatric cord HCTs, 129 HCTs (88.4%) were from unrelated donors. Due to the design of the survey, detailed analysis by diagnosis is limited to those centers declared as dedicated pediatric centers only. Here 123 centers reported a total of 4300 HCT in 3852 patients, of these 3191 HCT were allogeneic (74.2%) and 1109 (25.8%) autologous. Main indications for allogeneic HCT were AML (*n* = 433; 65% in early stage), ALL (*n* = 861; 50.5% in early stage), and NMD (*n* = 1372; 46.5%), of which 460 (33.5%) were for IEI. There were 1573 (53%) family and 1381 (46.8%) unrelated donor HCTs reported. Among the family donors, 745 (47.4%) were from a haploidentical relative. BM was used as the stem cell source in 1413 patients receiving an allogeneic HCT, of which 890 (56.6%) were family donors. Peripheral blood stem cells were used in 1436 patients with a slightly higher proportion seen in unrelated donors (*n* = 767; 53.4%) when compared to family donors (*n* = 669), and 105 were performed with CB. The main indications for autologous HCT were solid tumors (*n* = 762) and were predominantly for neuroblastoma (*n* = 383, 50.3%). Peripheral blood stem cells were used in the majority of autologous HCT (*n* = 889; 99%) with 9 patients receiving BM stem cells. No patients were reported as having received CB HCT. Non-myeloablative HCT was reported in 402 transplants and 265 patients received a DLI. The main reason for the DLI was graft enhancement or failure. A further 122 patients received IST for BM failure.

### Transplant activity rates by country in 2023

Assessing transplant rates per 10 million population (TR/10^7^) allowed the comparison of activity in countries adjusted for differences in population size. In the 2023 survey, the TR rates for allogeneic HCT within European countries only ranged from 2.7/10^7^ in Georgia to 537.9/10^7^ in Israel, followed by 422.8/10^7^ in Germany, 395.2/10^7^ in Belgium, 377.6/10^7^ in Lithuania, and 368.4/10^7^ in Switzerland. The median number of total allogeneic HCT by country was 132 and the median TR 148.4/10^7^. Four countries did not report any allogeneic HCT (Cyprus, Iceland, Latvia, and Luxembourg). For autologous HCT, rates ranged from 19.1/10^7^ in Kazakhstan to 629.4/10^7^ in Lithuania followed by 588.3/10^7^ in Italy, 575.8/10^7^ in Luxembourg, 559.2/10^7^ in the Netherlands, and 519.9/10^7^ in Norway. The median number of total autologous HCT by country was 185 and the median TR 259.8/10^7^. All countries participating in the annual survey reported doing autologous HCT [21].

### Immunosuppressive treatment in 2023

New in the survey this year was the possibility to report the number of patients treated with IST for BMF. One hundred and ninety-two centers reported a total of 736 patients having received IST in 2023. The majority of which were given to patients with aplastic anemia (*n* = 605), with a further 88 for patients with other BMFs.

### Advanced cellular therapy products (ATMPs) and DLI

Table 2 shows the number of patients who received advanced cellular therapy products or un-manipulated DLI infusions in 2023. The total number of patients receiving DLI infusion was 2875, an increase of +0.7% when compared to 2022. The main reported reason for treatment was relapse (*n* = 1299) and graft enhancement or failure (*n* = 686).

**Fig. 5:**
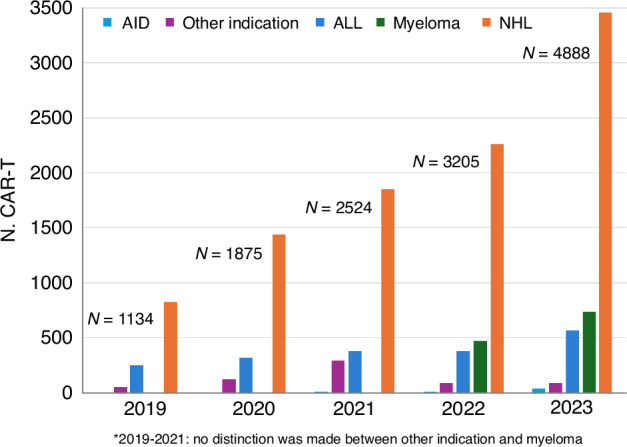
Increase in the number of patients receiving CAR-T therapy by main indication from 2019 to 2023.

A total of 6042 patients (+39.6% and 1713 therapies) received hematopoietic cellular therapies that qualified as medicinal products rather than cell transplants [19]. In 2023, the ongoing and impressive increase is continuing for gene-modified T cells, notably CAR-T cells, increasing from 301 reported in 2018 to 1134 in 2019, 1875 in 2020, 2524 in 2021, 3205 in 2022 and 4888 in 2023 (+52.5% since 2022) and an overall 16.2-fold increase (Fig. 5). Over these last 6 years, we observed that the number of patients treated with CAR-T cells increased constantly and did not seem to be impacted by the SARS-CoV-2 pandemic. The main indication for CAR-T cellular therapy since 2019 was B-NHL, increasing from 826 in 2019 to 3462 in 2023 and for ALL, the numbers increased from 252 in 2019 to 566 in 2023. The survey was adapted in recent years to capture data on other newly introduced disease indications for CAR-T cell treatment, such as myeloma which increased from 470 in 2022 to 736 in 2023 and other grouped indications (e.g., AID and solid tumors) where 124 were reported in 2023 (36 of which were for AID) [22–34]. In 2023, 258 centers in 30 countries reported CAR-T cell therapies. All centers reporting CAR-T cell therapies also reported experience in both allogeneic and autologous HCT with 233 centers reported having performed both allogeneic and autologous HCT, 18 autologous HCT only, and 7 centers performed allogeneic HCT only. Eighty-five patients receiving allogeneic CAR-T cell therapy were reported by 19 centers in 12 countries. For autologous CAR-T cell therapy, 4803 (98.3%) patients were reported in 250 centers in 30 countries. The median number of patients receiving CAR-T cell therapy reported by country was 47 (range 1–1160) and the median CAR-T cell rate was 44.7/10^7^, (range 0.1–297.1). The median number of patients receiving CAR-T cell therapy reported by country was 47 (range 1–1160) and the median CAR-T cell rate was 44.7/10^7^, (range 0.1–297.1). Rates ranged from 0.1/10^7^ in Iran to 297.1/10^7^ in Israel followed by 196.6/10^7^ in Switzerland, 137.5/10^7^ in Germany, 124.5/10^7^ in France, and 112.3/10^7^ in Spain. Two hundred and thirty-six of the 4888 patients receiving CAR-T cell therapy were from dedicated pediatric centers. Forty-six were allogeneic and 190 autologous (80.5%). The indications being, ALL (*n* = 220), NHL (*n* = 4), and other non-specified indications (*n* = 12).

The number of reported CAR-T cell therapies performed in Europe is increasing steadily and has reached a cumulative total of 13,927 patients treated between 2018 and 2023.

Other cellular therapies were reported in 1154 patients in 2023 from 219 centers in 34 countries. The most widely used was mesenchymal stromal cell therapy (*n* = 434; 96% allogeneic), their use being mainly to treat graft-versus-host disease. Promising results with mesenchymal stromal cell therapy have also been reported in AID. Numbers of non-CAR-T cellular therapy products have not greatly changed since 2019 with some increase in the use of MSC and NK cells. Of note, specific data on tumor-infiltrating lymphocytes is not collected separately in the annual survey.

## Discussion

The EBMT activity survey has been conducted annually since 1990 [1] and has amassed data on almost one million HCT procedures during this time. Over 47,500 transplants in almost 44,000 patients were reported in 2023. The largest number of transplants ever reported was in 2019 (48,412 in 43,581 patients). In 2020, a considerable decrease in transplant activity was observed, due to the SARS-CoV-2 pandemic. During the following 2 years, 2021–2022, we saw fluctuations in the numbers reported, possibly due to the ongoing effect of the pandemic and the slow recovery thereafter. Now, in 2023, we once again see an increase in activity in allogeneic HCT, reporting the highest number of transplants so far for allogeneic, but not for autologous HCT.

In autologous HCT there is a continued shift towards PCD now comprising 58% of the indications (Supplementary Fig. 1b), with a decrease in numbers for lymphoma most likely because these patients are now treated by CAR-T cell therapy. At this point, in time autologous HCT in myeloma is not competing with CAR-T therapy as CAR-T remains second-line therapy and autologous HCT remains mostly a first-line treatment in PCD. For AID, the overall decrease seen in 2020 based on EBMT Guidelines [22] is now gradually recovering to numbers similar to those prior to the pandemic.

In allogeneic HCT there is an increase in use mainly for myeloid malignancies, including AML but also MDS and MPN (Fig. 3). This contrasts with the stable use of allogeneic HCT for CML, over many years. This is possibly due to studies reporting good results for allogeneic HCT in AML, MDS, and MPN. The number of allogeneic HCT for CML declined steeply with the introduction of TKI in 2000 and has remained stable ever since at around 400 allogeneic HCTs per year for CML in the chronic phase as well as in more advanced disease. This can be interpreted as universal access to TKI having been reached around 2008 and patients transplanted thereafter receive treatment after TKI failure or intolerance, for the now rare situation of progressive disease on TKI or for primary blast crisis, i.e., transformation without prior exposure to TKI.

In allogeneic HCT, donor choice demonstrated a continuing trend of moving away from HLA-identical family donors, with the increasing use of unrelated donors and haploidentical donors. The decreased use of sibling donors in 2023, the number now being similar to that reported in 2006, is most likely due to a shift in preference from older family donors towards younger unrelated and possibly haploidentical donors based on studies comparing allogeneic HCT outcome between young unrelated or haploidentical donors and older sibling donors [35–38]. We do not have data on patient and donor age to analyze this further.

Most remarkable is the increase in the use of CAR-T cell therapy with a cumulative total of over 13,000 patients in Europe as of 2023. CAR-T treatment for myeloma is increasing rapidly but not as much as CAR-T for NHL (Fig. 5) and we see the first reports on CAR-T treatment for AID [32–34].

In summary, we see a full recovery from the post-pandemic times in the growth of allogeneic HCT surpassing the numbers reported prior to the pandemic in 2019 but not with autologous HCT where numbers remain lower than those reported in 2019. With autologous HCT, the indication of PCD is on the rise again but autologous HCT for lymphoma is declining. At the same time, we continue to see an impressive increase in the use of CAR-T cell therapy for NHL and PCD.

The annual activity survey of the EBMT reflects current activity and trends in the field of transplant technology. Ongoing studies using the EBMT benchmarking model with registry and survey data aim to assess the impact of international variation in activity and clinical practice across countries with similar and variable economies on survival outcomes. This report is valuable for the dissemination of the most recent information on indications, donor, and stem cell usage, which will ultimately be beneficial in health care planning.

## Supplementary information

Figure 1ab

Appendix of participating centers

## Data Availability

Datasets may be available upon request via EBMT Partnering (partnering@ebmt.org).
