# Supplementary material for: The 2023 EBMT report on hematopoietic cell transplantation and cellular therapies. Increased use of allogeneic HCT for myeloid malignancies and of CAR-T at the expense of autologous HCT
Source: Bone Marrow Transplant. 2025 Feb 12;60(4):519–28. doi: 10.1038/s41409-025-02524-2 (PMC11971038; doi:10.1038/s41409-025-02524-2)

Supplementary figure 1: relative proportion of disease indications for HCT in 2023.
Green shades: myeloid malignancies, blue: lymphoid malignancies, brown: solid tumors and red: non malignant disorders.

1a: allogeneic 1st HCT

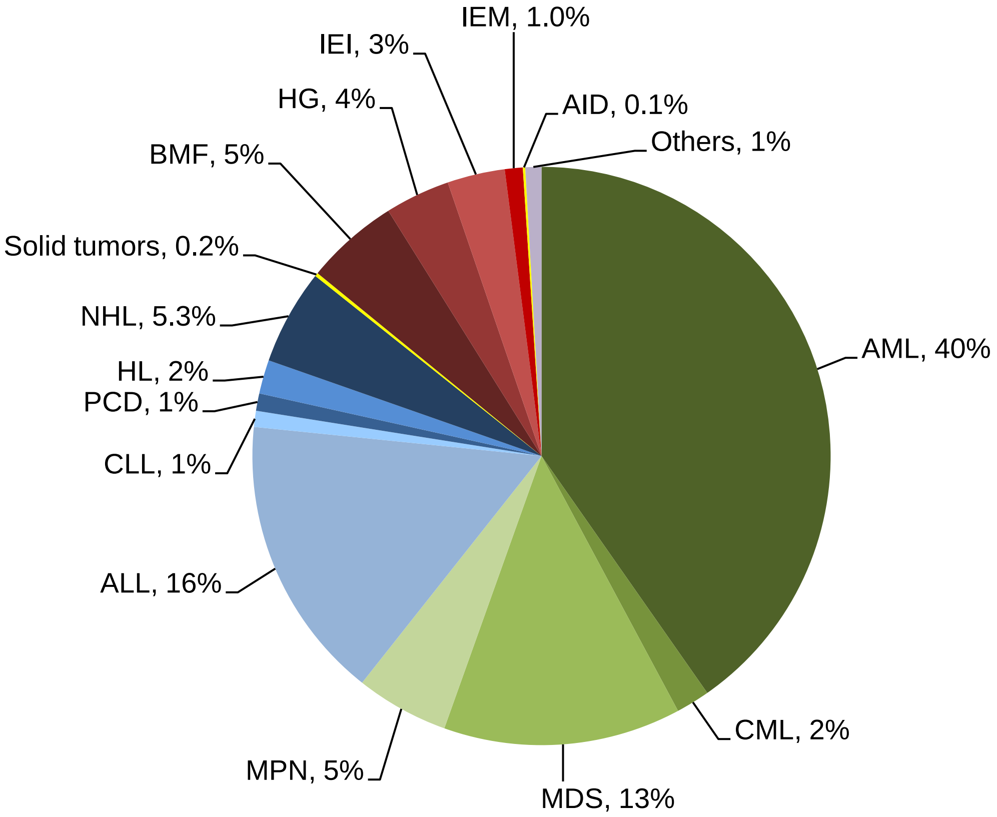

1b: autologous 1st HCT.

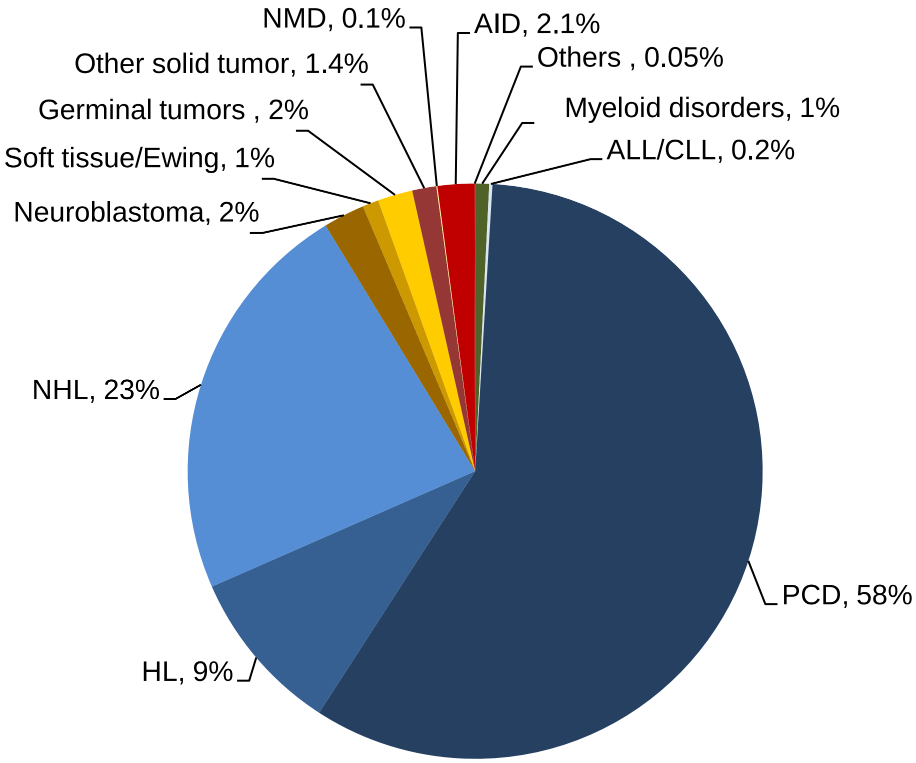

Supplement: Supplementary file 1 — Figure 1ab [file 41409_2025_2524_MOESM1_ESM.pdf]
