# Supplementary material for: The 2023 EBMT report on hematopoietic cell transplantation and cellular therapies. Increased use of allogeneic HCT for myeloid malignancies and of CAR-T at the expense of autologous HCT
Source: Bone Marrow Transplant. 2025 Feb 12;60(4):519–28. doi: 10.1038/s41409-025-02524-2 (PMC11971038; doi:10.1038/s41409-025-02524-2)
Supplement: Supplementary file 2 — Appendix of participating centers [file 41409_2025_2524_MOESM2_ESM.pdf]

APPENDIX 2023

EBMT Transplant Activity Survey: List of reporting transplant centers in 2023
(Number of reporting centers: total 1st HCT (total all HCT) N allogeneic 1st HCT / N autologous 1st HCT)

Europe 696 centers: (43,902 (47,731) 19,368 / 24,534

Algeria: (2 centers: 337 (350) 182/155)
Alger, Centre Pierre et Marie Curie, ads, peds, CIC 703, R. Hamladji (197 (210) 152/45)
Oran, University Hospital of Oran, ads, peds, CIC 993, N. Yafour, M. Brahimi, K. Amani, N.H. Hassam, M. Belabbes, N. Benali (140 (140) 30/110)

Armenia: (1 center: 46 (46) 4/42)
Yerevan, Hematology Centre R. H. Yeolyan, ads, peds, CIC 891, K. Meliksetyan (46 (46) 4/42)

Austria: (13 centers: 513 (563) 240/273)
Graz, University of Graz, ads, CIC 308, H. Greinix, H. Sill (66 (73) 42/24)
Graz, Universitäts Kinderklinik, peds, CIC 593, W. Schwinger, D. Sperl, M. Benesch, R. Dengg (6 (6) 4/2)
Innsbruck, University Hospital, ads, peds, CIC 271, D. Wolf, D. Nachbaur (67 (77) 30/37)
Klagenfurt, Klinikum Klagenfurt, ads, S. Eder (11 (11) 0/11)
Linz, Kepler University Hospital, ads, CIC 343, C. Schmitt (7 (8) 0/7)
Linz, AOKH der Elisabethinen, ads, CIC 594, J. Clausen, A. Weltermann (90 (105) 50/40)
Salzburg, LKA Salzburg, ads, CIC 356, R. Greil, G. Russ (18 (20) 0/18)
St. Pölten, University Hospital St. Pölten, ads, CIC 474, G. Krajnik, Ch. Fertl (23 (24) 0/23)
Vienna, Universitätsklinik fur Innere Medizin-AKH, ads, CIC 227, W. Rabitsch (108 (114) 90/18)
Vienna, St. Anna Kinderspital, peds, CIC 528, H. Pichler,W. Holter, S. Karlhuber (26 (34) 24/2)
Vienna, Hanusch- Krankenhaus, ads, CIC 743, F. Keil (52 (52) 0/52)
Vienna, Donauspital, ads, CIC 767, C. Sebesta, P. Kier, C. Zhang (10 (10) 0/10)
Vienna, Kilinik Ottakring, Wilhelminenspital, ads, CIC 828, W. Hilbe, M. Schreder (29 (29) 0/29)

Azerbaijan: (1 center: 20 (20) 19/1)*
Baku, Republican Thalassemia Centre, peds, P. Safarova, N. Aliyeva Rafiq, S. Saila Rauf (20 (20) 19/1)* late report
Ganja, Ganja International Hospital, ads, S. Dincer, I. Zeynep Bulut (no report)

Republic of Belarus: (2 centers: 185 (186) 51/134)
Minsk, Scientific Centre of Surgery, Transplantology and Hematology, ads, N. Milanovich (148 (148) 22/126)
Minsk, Belorussian Center, peds, CIC 591, Y. Mareika, N. Minakovskaya, D. Prudnikov, N. Kirsanova, M. Naumovich, L. Zherko, O. Mishkova, A. So (37 (38) 29/8)

Belgium: (16 centers: 882 (965) 433/449)
Antwerp, Stuivenberg ZH, ads, CIC 339, W. Ka Lung, D. Breems (26 (29) 14/12)
Antwerp, University Antwerpen, ads, CIC 996, A. Gadisseur (56 (56) 25/31)
Brugge, AZ Sint-Jan Brugge AV, ads, CIC 506, T. Lodewyck, A. Schauwvlieghe, S. Snauwaert, J. Van Droogenbroeck (74 (74) 46/28)
Brussels, Institute Jules Bordet and the Childrens Hospital, ads, peds, CIC 215, N. Meuleman, C. Devalck (118 (125) 71/47)
Brussels, Clinique Universitaire St. Luc, ads, peds, CIC 234, X. Poiré (78 (86) 44/34)
Brussels, University Hospital, ads, CIC 630, A. de Becker, N. de Beule, F. Trullemans (38 (48) 13/25)
Charleroi, Grand Hôpital de Charleroi Notre-Dame, ads, CIC 349, D. Pranger (11 (13) 11/0)
Gent, University Hospital, ads, peds, CIC 744, C. Dhooge, T. Kerre, V. Bordon (111 (127) 51/60)
Haine St. Paul, Hôpital de Jolimont, ads, CIC 234, A. Sperone, F. Van Obbergh, H. Petre (18 (20) 0/18)
Hasselt, Jessa Ziekenhuis, ads, CIC 632, K. Theunissen (34 (36) 0/34)
Leuven, University Hospital Gasthuisberg and Leuven, ads, peds, CIC 209, J. Maertens, H. Schoemans, A. Uyttebroeck, M. Renard (145 (153) 75/70)
Liège, University Hospital Sart-Tilman, ads, peds, CIC 726, F. Baron, B. de Prijck, J. Caers (78 (84) 41/37)
Roeselare, AZ Delta vzw, ads, CIC 646, D. Deeren (30 (33) 15/15)

Turnhout, AZ Turnhout, ads, CIC 910, I. Vrelust, K. Voet (12 (12) 0/12)
Wilrijk-Antwerp, St. Agustinus Hospital, ads, CIC 715, J. Lemmens, C. Schuermans (7 (13) 0/7)
Yvoir, Clinique universitaire de Mont-Godinne, ads, CIC 234, C. Doyen (46 (56) 27/19)

Bosnia-Herzegovina: (no report)
Sarajevo, Clinical Centre, ads, A. Sofo-Hafizovic (no report)
Tuzla, University Clinical Centre, ads, H. Sahovic , S. Hasić, A.Jahić, V. Simendić (no report)

Bulgaria: (2 centers: 89 (99) 31/58)
Sofia, University Hospital Queen Johanna-Isul, peds, CIC 346, D. Konstantinov, B. Avramova (0 (0) 0/0)
Sofia, National Centre of Hematology, ads, CIC 859, G. Kondeva, G. Mihaylov, K.Venkov, A. Bankova, V. Nikolova, Y. Stefanova, I.Tonev, M. Minchev, S. Si (89 (99) 31/58)

Croatia: (3 centers: 189 (218) 82/107)
Split, University Hospital Split, ads, CIC 1033, D. Lozic (19 (19) 0/19)
Zagreb, University Hospital Rebro, ads, peds, CIC 302, R. Vrhovac, R. Serventi-Seiwerth, N. Duraković, L. Desnica, A. Vulić, I. Aurer, B. Dreta, E. Bilić (121 (138) 82/39)
Zagreb, University Hospital Dubrava, ads, CIC 407, V. Pejsa, O. Jaksic, Z. Prka (49 (61) 0/49)
Zagreb, Hospital Merkur, ads, CIC 466, D. Radic-Kristo, V. Zatezalo (no report)

Cyprus: (1 center: 30 (30) 0/30)
Nicosia, Nicosia General Hospital, ads, CIC 575, M. Antoniades , C. Stylianou (30 (30) 0/30)

Czech Republic: (9 centers: 586 (697) 231/355)
Brno, Masaryk University Hospital, ads, peds, CIC 597, T. Kepak, F. Folber (110 (129) 35/75)
Hradec Kralové, Charles University Hospital, ads, CIC 729, P. Zak (69 (80) 25/44)
Olomouc, University Hospital, ads, CIC 574, L. Raida, I. Skoumalova, T. Szotkowski, A. Kuba, M. Cernan, A. Hruskova, A. Kredatusova, I. Maresova (47 (54) 21/26)
Ostrava, University Hospital Ostrava, ads, CIC 156, Z. Koristek, I. Tvrda, R. Hajek (52 (62) 19/33)
Pilsen, Charles Hospital, ads, CIC 718, P. Jindra, A. Jungova (97 (117) 33/64)
Prague, Charles University Hospital, ads, CIC 318, T. Kozak (31 (31) 0/31)
Prague, University Hospital Motol, peds, CIC 452, P. Sedlacek (42 (56) 31/11)
Prague, Institute of Hematology and Blood Transfusion, ads, CIC 656, A. Vitek (67 (73) 67/0)
Prague, Charles University, ads, CIC 745, M. Trneny (71 (95) 0/71)

Denmark: (3 centers: 334 (362) 164/170)
Aalborg, Aalborg Hospital, ads, CIC 848, A. Svirskaite, S. Ramme Bro, G. ThomsenT. Haunstrup (28 (28) 0/28)
Aarhus, Aarhus Amtssygehus, ads, peds, CIC 634, C. Juhl-Christensen, I. Petruskevicius (107 (121) 53/54)
Copenhagen, Rigshospitalet, ads, peds, CIC 206, H. Sengelov, M. Ifversen (199 (213) 111/88)

Estonia: (2 centers: 75 (84) 19/56)
Tallinn, North Estonia Medical Centre, ads, CIC 984, K. Palk (41 (46) 0/41)
Tartu, University Hospital, ads, peds, CIC 746, A.Kaare (34 (38) 19/15)

Finland: (7 centers: 359 (372) 146/213)
Helsinki, Children's Hospital, peds, CIC 219, S. Ryhänen (30 (30) 29/1)
Helsinki, Helsinki University Central Hospital, ads, CIC 515, U. Salmenniemi (107 (108) 63/44)
Helsinki, Helsinki University Hospital, ads, CIC 833, S. Mannisto (21 (21) 0/21)
Kuopio, University Hospital, ads, CIC 396, A. Partanen (44 (46) 0/44)
Oulu, Oulu University Central Hospital, ads, CIC 690, M. Säily (29 (29) 0/29)
Tampere, University Hospital, ads, peds, CIC 635, M. Sankelo, M. Sinisalo (44 (48) 0/44)
Turku, University Central Hospital, ads, CIC 225, M. Itälä-Remes, M. Putkonen, P. Ettala, E. Lauraeus-Käkelä, H. Järviö, J. Raali, R. Mäkinen, J. Pako (84 (90) 54/30)

France: (70 centers: 4353 (4703) 2058/2295)
Amiens, CHU d'Amiens, ads, CIC 955, A. Charbonnier, J.P. Marolleau (100 (100) 65/35)
Angers, Centre Hospitalier, ads, CIC 650, M. Hunault-Berger, S. Francois (63 (63) 29/34)
Argenteuil, Hopital Victor Dupouy, ads, CIC 199, A. Al Jijakli (17 (18) 0/17)

Arras, Groupe Hospitalier Artois Ternois, ads, CIC 999, P. Lionne-Huyghe (9 (9) 0/9)
Bayonne, C.H. De la Cote Basque, ads, CIC 875, S. Labarrere, A. Banos (37 (40) 0/37)
Besancon, Hopital Jean Minjoz and St.Jacques, ads, peds, CIC 233, E.Deconinck (87 (89) 49/38)
Bordeaux, CHU Hopitalier Pellegrin- Enfants, peds, CIC 978, C. Jubert, M. Angoso (37 (46) 21/16)
Brest, Hopital Morvan, CHU de Brest, ads, peds, G. Guillerm, M.A. Couturier, E. Follet, J. Kerebel (63 (63) 25/38)
Caen, CHU Caen Institut d'hématologie de Basse-Normandie CHU, ads, CIC 251, G. Damaj,S. Chantepie (89 (92) 41/48)
Clermont Ferrand, CRCTCP, CHU Estaing, ads, CIC 273, J.O. Bay, G. Piquemal (83 (90) 50/33)
Clermont Ferrand, CRCTCP, CHU Estaing, peds, CIC 273, J. Kanold, E. Rochette (8 (9) 6/2)
Colmar, Louis Pasteur Hospital, ads, J. Chapiro (no report)
Corbeil Essonne, Hopital Gilles de Corbeil, peds, B. Joly,S. Haiat (28 (29) 0/28)
Créteil, Hopital Henri Mondor, ads, CIC 252, C. Cordonnier, S. Maury (43 (44) 43/0)
Créteil, Hopital Henri Mondor, ads, CIC 432, F. Lemonnier, R. Gounot (38 (38) 0/38)
Dijon, Hopital des Enfants, ads, CIC 667, D. Caillot (no report)
Dunkerque, Centre Hospitalier, ads, CIC 964, C. Fruchart, M. Wetterwald (18 (20) 0/18)
Grenoble, CHV Grenoble Alpes, ads, peds, CIC 270, C.E. Bulabois, M. Carré, A. Thiebaut (98 (109) 49/49)
La Réunion, CHU Felix Guyon, Saint Denis Centre, ads, E. Randriamalala (17 (18) 0/17)
La Réunion, St. Pierre CHU Sud La Réunion, ads, CIC 404, C. Mohr (47 (50) 11/36)
Le Chesnay, Hôpital André Mignot, Varsailles, ads, CIC 877, P.H. Rousselot, A.M. Ngo Nloga (34 (38) 0/34)
Lens, Service d'Hématologie, ads, CIC 874, C. Bories, L. Stalnikiewicz (15 (15) 0/15)
Lille, Hopital Claude Huriez, ads, CIC 277, I. Yakoub-Agha (136 (143) 109/27)
Lille, Hopital Jeanne de Flandre, peds, CIC 963, B. Bruno, B. Nelken (23 (23) 23/0)
Lille, Centre Oscar Lambret, ads, peds, CIC 972, A. Defachelles (16 (22) 0/16)
Lille, Centre Hospitalier Saint Vincent, ads, CIC 991, B. Carpentier, L. Pascal (no report)
Limoges, CHU Dupuytren, ads, CIC 977, P. Turlure, J. Abraham (38 (41) 17/21)
Lyon, Centre Léon Bérand, ads, CIC 241, E. Nicolas-Virelizier (43 (54) 0/43)
Lyon, Centre Hospitaier Lyon Sud, ads, CIC 671, H. Labussiere-Wallet (100 (102) 100/0)
Lyon, Institue d'Hématologie et d'Oncologie Pédiatrique, peds, CIC 806, C. Renard (39 (46) 26/13)
Lyon, Hospices Civils de Lyon, ads, CIC 901, H. Ghesquieres (no report)
Marseille, Institut Paoli I. Calmettes, ads, CIC 230, D. Blaise, C. Chabannon (228 (247) 136/92)
Marseille, Hopital Timone Enfants, peds, CIC 301, G. Michel, C. Coze (52 (54) 37/15)
Marseille Bouches du Rhone, Centre Hospitalier Universitaire La Conception, ads, R. Costello (16 (16) 0/16)
Meaux, CHU de Meaux, ads, CIC 194, J. Frayfer, W. Abarah (16 (17) 0/16)
Montpellier, CHR Lapeyronie and CHU Arnaud de Villeneuve, ads, peds, CIC 926, N. Fegueux, A. Sirvent (181 (188) 83/98)
Mulhouse, Hopital E. Muller, ads, CIC 944, B. Drénou, M. Ojeda-Uribe (21 (23) 0/21)
Nantes, CHU Nantes, ads, peds, CIC 253, P. Chevallier, F. Rialland (195 (207) 87/108)
Nice, Hopital de l'Archet, ads, CIC 523, T. Cluzeau, M.Loschi, P. Rohrlich, (66 (68) 52/14)
Nice, Centre Antoine Lacassagne, ads, CIC 973, L. Gastaud (30 (32) 0/30)
Orleans, CHR Orléans, ads, CIC 164, M. Alexis, C. Benbrahim (20 (23) 0/20)
Paris, Hôpital Cochin, ads, D. Bouscary (29 (39) 0/29)
Paris, Hôpital Necker, ads, peds, CIC 160, O. Hermine, F. Suarez (43 (49) 30/13)
Paris, Hôpital Neckar des enfants malades, peds, CIC 201, B. Neven, S. Blanche (43 (44) 41/2)
Paris, Hôpital St. Louis, ads, peds, CIC 207, G. Socié, M. Robin, R Peffault de La Tour (142 (149) 134/8)
Paris, Hôpital St. Louis, ads, CIC 805, C. Thieblemont (15 (15) 0/15)
Paris, Immuno-Hèmatologie, Hôpital St. Louis, ads, CIC 969, B. Arnulf (67 (67) 0/67)
Paris, Hôpital Pitié Salpétière, ads, CIC 262, S. Nguyen Quoc, S. Choquet (117 (127) 52/65)
Paris, Hôpital Robert Debré, peds, CIC 631, J.H. Dalle (47 (49) 47/0)
Paris, Clarmart, Hopital d'Instruction des Armées Percy, ads, peds, CIC 665, JV. Malfuson (60 (63) 32/28)
Paris, Hopital d'enfants Armand Trousseau, peds, CIC 675, G. Leverger, A. Petit (5 (7) 0/5)
Paris, Institut Curie, peds, CIC 702, Ph. Brault (14 (22) 0/14)
Paris, Hôpital St. Antoine, ads, CIC 775, M. Mohty, S. Lapusan (102 (102) 56/46)
Pessac, Hôpital du Haut Leveque, CHU Bordeaux, ads, CIC 267, N. Milpied, E. Forcade (201 (213) 102/99)

Poitiers, CHU de Poitiers, Hôpital La Miletrie, ads, peds, CIC 264, X. Leleu, M. Maillard, (120 (130) 54/66)
Pontoise, Hospital René Dubos, ads, I. Vaida, R. Benramdane, S. Sarghi, K. Ouis-Tarhi, H. Gonzalez (19 (19) 0/19)
Reims, Hop. Robert Debré, ads, CIC 959, A. Quinquenel, C. Himberlin, A. Berceanu, Q.H. Le (26 (27) 0/26)
Rennes, CHU Rennes, ads, CIC 661, T. Lamy, M Bernard (120 (129) 49/71)
Rennes, Clinique Médical Infantil, CHRU, peds, CIC 661, V. Gandemer (26 (28) 20/6)
Roubaix, Hopital V. Provo, ads, CIC 968, M. Wémeau, J. Hieulle, I. Dervite, S. Dennetiere, L. Dumeignil (12 (13) 0/12)
Rouen, Hopital Charles Nicolle, peds, CIC 932, P. Schneider, N. Buchbinder (7 (7) 7/0)
Rouen, Centre Henri Becquerel, ads, CIC 941, H. Tilly, N. Contentin (88 (96) 38/50)
Saint Priest en Jarez, Institut de Cancérologie Lucien Neuwirth, ads, CIC 250, D. Guyotat, J. Cornillon (61 (64) 29/32)
Saint Quentin, Centre Hospitalier De Saint Quentin, ads, CIC 406, R. Garidi (8 (9) 0/8)
St. Cloud, Institut Curie, Hopital René Huguenin, ads, C. Soussain (48 (48) 0/48)
Strasbourg, ICANS, ads, peds, CIC 672, B. Lioure, C. Paillard, Y.Meziane, R. A. Ceredig, (120 (123) 60/60)
Toulouse, Institut Universitaire du Cancer Toulouse Oncopole, ads, CIC 624, A. Huynh, C. Recher (164 (183) 73/91)
Toulouse, Hopital Purpan, peds, CIC 952, M. Gambart, L. Olivier, M. Pasquet, C. Theron, G. Alphonsa, C. Fabaron (13 (20) 0/13)
Tours, Hôpital Bretonneau, ads, CIC 272, E. Gyan, A.Villate, S.Ondet (96 (96) 35/61)
Troyes, Centre Hospitalier de Troyes, ads, CIC 472, A. Santagostino (30 (37) 0/30)
Valenciennes, Centre Hosp. de Valenciennes, ads, S. Tricot (32 (32) 0/32)
Vandoeuvre-les-Nancy, CHU Brabois and Hôpital d'Enfants, ads, peds, CIC 676, M. Rubio, P. Feugier, A. Campidelli, C. Pochon (111 (137) 67/44)
Villejuif, Institut Gustave Roussy, peds, CIC 503, C. Rigaud (34 (55) 0/34)
Villejuif, Gustave Roussy Cancer Campus, ads, CIC 666, C. Castilla-Llorente (182 (218) 73/109)

Georgia: (1 center: 74 (74) 1/73)
Tbilisi, High Technology Medical Center, ads, G. Ingorokva (74 (74) 1/73)

Germany: (124 centers: 6922 (7895) 3327/3595)
Aachen, Universitätsklinikum, ads, CIC 348, T. Brümmendorf, E. Jost (87 (98) 41/46)
Aachen, Universitätsklinikum, peds, CIC 348, U. Kontny (8 (8) 8/0)
Augsburg, Universitätsklinikum, ads, CIC 152, C. Schmid (76 (90) 32/44)
Bad Saarow, HELIOS-Klinikum, ads, R. Ratei (9 (13) 0/9)
Bamberg, Klinikum am Bruderwald, ads, CIC 488, R. Seggewiss-Bernhardt (12 (19) 0/12)
Bayreuth, Klinikum Bayreuth, ads, A. Kiani (15 (18) 0/15)
Berlin, Vivantes Klinikum Neukoelln, ads, M de Wit, M. Fausta Ribezzo (29 (29) 0/29)
Berlin, Charité, Campus Virchow Klinikum, ads, CIC 807, I. Blau (250 (305) 125/125)
Berlin, Charité, Campus Virchow Klinikum, peds, CIC 336, A. von Stackelberg (39 (43) 35/4)
Berlin, HELIOS Klinikum Berlin Buch, ads, peds, CIC 518, J. Niederland (103 (105) 56/47)
Bielefeld, Evangelisches Klinikum Bethel, ads, CIC 116, F. Weissinger, E.B. Zinngrebe (17 (20) 0/17)
Bielefeld, Klinikum Bielefeld, ads, CIC 949, M. Görner, S. Probst (18 (24) 0/18)
Bochum, Knappschaftskrankenhaus, ads, CIC 124, R. Schroers, T. Mika (130 (148) 48/82)
Bonn, Universitätsklinikum, peds, D. Dilloo (6 (6) 3/3)
Bonn, Johanniter-Krankenhaus und Waldkrankenhaus, ads, Y.-D. Ko, H.C. Orfgen, R. Forkert, M.Monin, J. Reinke (34 (36) 0/34)
Bonn, Universitätsklinikum, ads, CIC 134, P. Brossart, T. Holderried (77 (79) 49/28)
Braunschweig, Städtisches Klinikum, ads, CIC 674, J. Krauter, M. Ahlborn (48 (60) 0/48)
Bremen, Evangelisches Diakonie-Krankenhaus GmbH, ads, CIC 111, R.U.Trappe, K. Schmitz (21 (27) 0/21)
Bremen, Klinikum Bremen-Mitte, ads, peds, CIC 602, B.Hertenstein, S. Kaun (35 (46) 9/26)
Chemnitz, Klinikum Chemnitz GmbH, ads, CIC 104, M. Hänel, A.Morgner (57 (62) 15/42)
Cottbus, Carl-Thiem-Klinikum, ads, CIC 102, M. Schmidt-Hieber (8 (9) 0/8)
Darmstadt, Klinikum Darmstadt GmbH, ads, CIC 1065, C. Schwebel-Kottke (20 (20) 0/20)
Dortmund, St. Johannes Hospital, ads, CIC 125, R. Meyer (55 (61) 27/28)

Dresden, Universitätsklinikum Carl Gustav Carus, ads, CIC 808, M. Bornhäuser, J. Schetelig (150 (178) 81/69)
Dresden, Universitätsklinikum Carl Gustav Carus, peds, CIC 808, R. Berner, M. Polacik (11 (14) 5/6)
Duisburg, HELIOS Klinikum, ads, CIC 519, G. I. Grigoleit, U. Wieschermann (49 (60) 20/29)
Düsseldorf, Heinrich Heine Universitätsklinikum, ads, CIC 390, G. Kobbe (150 (173) 92/58)
Düsseldorf, Universitätsklinikum, peds, CIC 651, A. Borkhardt, R.Meisel (33 (42) 25/8)
Erfurt, Helios-Klinikum, ads, CIC 966, H. Sayer, F. Schreiber, A.-K. Langhammer, M. Scheibe, K. Gräger (28 (39) 0/28)
Erlangen, Universitäts Klinik für Kinder und Jugendliche, peds, CIC 809, J. Wölfle, N. Naumann-Bartsch (10 (10) 9/1)
Erlangen, Universitätsklinikum, ads, CIC 809, A. Mackensen, J. Winkler (77 (83) 43/34)
Essen, West German Cancer Center, ads, M. Schuler, S. Bauer (8 (8) 0/8)
Essen, Universitätsklinikum, ads, CIC 259, T. Schroeder, C. Rautenberg, C. Reinhardt, B. von Tresckow, R. Noppeney, (198 (211) 126/72)
Essen, Universitätsklinikum, peds, CIC 259, D. Reinhardt, S. Schönberger, M. Höfs, O. Basu (27 (34) 20/7)
Essen, Evangelisches Krankenhaus Essen-Werden GmbH, ads, CIC 784, P.Reimer, M. Dürholt (57 (60) 15/42)
Flensburg, St. Franziskus Hospital, ads, CIC 970, A. Krackhardt (20 (22) 7/13)
Frankfurt, Universitätsklinikum d. J. W. von Goethe Universität, peds, CIC 138, P.Bader, J.-H. Klusmann (37 (40) 34/3)
Frankfurt, Klinikum Frankfurt Oder GmbH, ads, CIC 190, M. Kiehl (30 (38) 21/9)
Frankfurt, Krankenhaus Bethanien, ads, CIC 193, W. Knauf (23 (23) 0/23)
Frankfurt, Universitätsklinikum d. J. W. Goethe Universität, ads, CIC 297, G. Bug, H. Serve (132 (145) 87/45)
Freiburg, Universitätsklinikum, ads, CIC 810, J.Duyster, R. Zeiser, M.Engelhardt (205 (218) 111/94)
Freiburg, Universitatsklinikum, peds, CIC 810, T. Feuchtinger, B. Strahm (33 (38) 31/2)
Georgsmarienhütte, Niels Stensen Kliniken Franziskus Hospital Hardenberg, ads, CIC 1044, R. Peceny, J. Atzpodien (21 (26) 0/21)
Giessen, Universitätsklinikum, ads, CIC 463, M. Rummel (14 (14) 0/14)
Giessen, Universitätsklinikum, peds, CIC 326, C. Mauz-Körholz (7 (7) 7/0)
Göttingen, Universitätsklinikum, ads, CIC 552, G. Wulf (126 (136) 74/52)
Greifswald, Universitätsklinikum, ads, CIC 530, W. Krüger (54 (64) 33/21)
Greifswald, Universitätsklinikum, peds, CIC 908, H. Lode, K. Ehlert (14 (14) 8/6)
Hagen, St. Josefs Hospital, ads, CIC 536, D. Kraemer (22 (26) 0/22)
Halle, Universitätsklinikum, ads, CIC 338, L. Müller, K. Lauer (70 (85) 41/29)
Halle, Universitätsklinikum, peds, CIC 654, J. Höll, K.Kafa (6 (6) 5/1)
Hamburg, Asklepios Klinik St. Georg, ads, CIC 153, A. Elmaagacli (90 (92) 55/35)
Hamburg, Asklepios Klinik Altona, ads, CIC 366, H. Salwender (26 (31) 0/26)
Hamburg, Universitätsklinikum Eppendorf, ads, CIC 614, N.Kröger (217 (230) 190/27)
Hamburg, Unversitätsklinikum Eppendorf, peds, CIC 882, I. Müller (47 (51) 40/7)
Hamburg, Universitätsklinikum Eppendorf - Onkologisches Zentrum, ads, CIC 673, C. Bokemeyer, K. Weisel, W. Alsdorf, I. Boehlke (101 (129) 0/101)
Hamm, St. Barbara-Klinik, ads, CIC 470, H. Duerk, A. S. Hilbig-Cordes (17 (24) 0/17)
Hamm, Evangelisches Krankenhaus, ads, CIC 509, A. Stoltefuss (7 (7) 0/7)
Hannover, Klinikum Siloah, ads, D. Dörfel (16 (23) 0/16)
Hannover, Medizinische Hochschule, ads, CIC 295, M. Eder (117 (123) 83/34)
Hannover, Medizinische Hochschule, peds, CIC 295, C. Kratz, M. Sauer (28 (32) 24/4)
Heidelberg, Universitätsklinikum, ads, CIC 524, C. Müller-Tidow, P. Dreger (263 (331) 117/146)
Heidelberg, Angelika-Lautenschläger-Klinik, peds, CIC 524, A. Kulozik, J. Greil (12 (12) 9/3)
Homburg/Saar, Universität des Saarlandes, ads, CIC 785, L. Thurner, M. Bewarder, J. Bittenbring, U. Kayser (101 (119) 46/55)
Homburg/Saar, Universität des Saarlandes, peds, CIC 785, M. Remke, T. Kren, D. Schöndorf (1 (1) 0/1)
Jena, Universitätklinikum, ads, CIC 533, I. Hilgendorf, A. Hochhaus (95 (104) 37/58)
Jena, Universitätsklinikum, peds, CIC 750, H. Proquitté, K. Kentouche (11 (11) 9/2)
Kaiserslautern, Westpfalzklinikum, ads, CIC 357, G. Held (9 (9) 0/9)
Karlsruhe, Städtisches Klinikum, ads, CIC 290, M.Bentz, M. Ringhoffer (56 (65) 26/30)
Kassel, Klinikum Kassel, ads, peds, CIC 118, M. Wolf, B. Ritter, M. Nathrath (25 (27) 0/25)
Kassel, Gesundheit Nordhessen, Klinikum Kassel, peds, CIC 118, M. Nathrath, M. Rodehueser (2 (2) 0/2)

Kiel, Universitätsklinikum Schleswig-Holstein, ads, CIC 256, F. Stölzel (83 (90) 53/30)
Kiel, Universitätsklinikum Schleswig-Holstein, peds, CIC 256, M. Schrappe, G. Cario (13 (15) 9/4)
Kiel, Städtisches Krankenhaus, ads, CIC 895, R. Repp (12 (14) 0/12)
Koblenz, Gemeinschaftsklinikum Mittelrhein, ads, CIC 879, D.Niemann (22 (22) 0/22)
Köln, Universitätsklinikum, ads, CIC 534, M. Hallek,Ch. Scheid, T. Simon (191 (213) 80/111)
Köln, Universitätsklinikum Köln, peds, CIC 534, T. Simon, P. Landgraf (7 (7) 0/7)
Leipzig, Universitätsklinikum, ads, CIC 389, U. Platzbecker (134 (152) 68/66)
Leipzig, Universitätsklinikum, peds, CIC 389, H. Christiansen, J.-S. Kühl (15 (15) 15/0)
Lemgo, Klinikum Lippe, ads, S. Bagdonas, P. Breuch (11 (18) 0/11)
Lübeck, Universitätsklinikum Schleswig Holstein, ads, CIC 367, F. Wortmann (56 (62) 25/31)
Lübeck, Universitätsklinikum Schleswig Holstein, peds, CIC 367, T. Langer (0 (0) 0/0)
Ludwigshafen, Klinikum der Stadt, ads, M. Hoffmann, S. Kapp-Schwoerer, C. Drozdz, K. Ast-Maciol, P. Warnecke (13 (14) 0/13)
Magdeburg, Universitätsklinikum, ads, CIC 359, D. Mougiakakos, D. Wolleschak (56 (66) 33/23)
Mainz, Universitätsklinikum, ads, CIC 786, E. Wagner-Drouet, M. Theobald (166 (182) 115/51)
Mannheim, Universitätsklinikum, ads, CIC 142, W K. Hofmann, S. Klein (53 (59) 33/20)
Marburg, Universitätsklinikum, ads, CIC 645, A. Neubauer, A. Burchert (118 (139) 62/56)
Meschede, Klinikum Hochsauerland GmbH, ads, CIC 489, M. Wattad, E. Lange (22 (22) 0/22)
Minden/Westfalen, Klinikum Minden, ads, CIC 113, H.-J. Tischler, K. Wille (19 (22) 0/19)
Möchengladbach, Kliniken Maria Hilf GmbH, ads, U.Graeven (5 (5) 0/5)
Munich, Klinikum Schwabing, ads, A. Hausmann (54 (55) 25/29)
Munich, Klinikum Schwabing, peds, CIC 189, J. Hauer, I. Teichert von Lüttichau (20 (22) 13/7)
Munich, von Haunersches Kinderspital, peds, CIC 513, M. Albert, T. Vallee (33 (35) 26/7)
Munich, Universitätsklinikum Grosshadern, ads, CIC 513, J.Tischer (79 (83) 51/28)
Munich, Klinikum Rechts der Isar, ads, CIC 558, M. Verbeek (110 (127) 52/58)
Munich, Rotkreuz Klinikum, ads, CIC 883, M. Hentrich (65 (87) 0/65)
Münster, Universitätklinikum, ads, CIC 680, M. Stelljes (226 (250) 125/101)
Münster, Universitätklinikum Münster, peds, CIC 505, C. Rössig, B. Burkhardt (33 (38) 27/6)
Nürnberg, Klinikum Nürnberg Nord, ads, CIC 625, S Knop, K. Schäfer-Eckart (91 (107) 32/59)
Oldenburg, Universitätsklinikum, ads, CIC 749, CH. Köhne, C. Kimmich, J. Caspar (76 (88) 31/45)
Osnabrück, Klinikum Osnabrück, ads, CIC 101, C. Petz, I. Hussein Mohamed (12 (13) 0/12)
Paderborn, Brüderkrankenhaus St. Josef, ads, CIC 936, T. Gaska (13 (13) 0/13)
Potsdam, Klinikum Ernst von Bergmann, ads, CIC 106, K. Jordan, F. Breywisch, S. Hyeon Kim (28 (33) 0/28)
Regensburg, Universitätsklinikum, ads, CIC 787, M. Edinger, D. Wolff, S. Raithel (133 (149) 57/76)
Regensburg, Universitätsklinikum, peds, CIC 787, S. Corbacioglu, J. Föll (19 (22) 15/4)
Rostock, Universitätsklinikum, ads, CIC 585, C. Junghanss, J. Lakner (60 (79) 26/34)
Rotenburg/Wümme, Agaplesion Diakonieklinikum, ads, A. Sabitzer, A. Meinhardt (11 (11) 0/11)
Schwäbisch Hall, Diakonie-Klinikum, ads, CIC 1062, M. Medinger (8 (8) 0/8)
Schwerin, Helios Kliniken Schwerin, ads, CIC 447, T. Bartscht (2 (2) 0/2)
Siegen, St. Marien-Krankenhaus, ads, CIC 135, S. Hain, R. Naumann (10 (13) 0/10)
Siegen, Diakonie Klinikum GmbH, ads, CIC 1069, R. Naumann, M. Klump, R. Marzell (2 (2) 0/2)
Stuttgart, Klinikum Stuttgart, Katharinenhospital, ads, CIC 143, G. Illerhaus (51 (59) 13/38)
Stuttgart, Robert Bosch Krankenhaus, ads, CIC 145, H.-G. Kopp, M. Kaufmann, S. Martin (65 (83) 24/41)
Stuttgart, Diakonie-Klinikum, ads, CIC 146, J. Greiner, S. Jung (45 (56) 25/20)
Stuttgart, Universitätsklinikum Olgahospital, peds, CIC 701, C. Blattmann, H.-M. Teltschik (5 (5) 0/5)
Tübingen, Universitätsklinikum, ads, CIC 223, C. Lengerke, W. Bethge (142 (169) 73/69)
Tübingen, Universitätsklinikum, peds, CIC 535, P.Lang (38 (45) 29/9)
Ulm, Universitätsklinikum, ads, CIC 204, H. Döhner, E. Sala, D. Bunjes (179 (207) 86/93)
Ulm, Kinderklinik Universitätsklinikum, peds, CIC 204, A.Schulz (27 (31) 22/5)
Villingen-Schwenningen, Schwarzwald-Baar Klinikum, ads, P. La Rosée (21 (27) 0/21)
Wiesbaden, Dr. Horst Schmidt Kliniken, ads, CIC 586, W. Blau, A. Brecht (20 (25) 0/20)
Winnenden, Rems-Murr Kliniken, ads, CIC 180, M. Schaich (9 (12) 0/9)
Würzburg, Universitätsklinikum, peds, CIC 196, P-G. Schlegel, M. Wölfl (20 (30) 14/6)
Würzburg, Universitätsklinikum, ads, CIC 712, H.Einsele, D. Teschner (212 (229) 89/123)

Greece: (13 centers: 405 (436) 209/196)
Alexandroupolis, Thrace University Med. School, ads, I. Kotsianidis (0 (0) 0/0)
Athens, G. Gennimatas Hospital, ads, G. Gkortzolidis (2 (2) 0/2)

Athens, Athens Medical Center, ads, CIC 603, A. Pigaditou (6 (6) 0/6)
Athens, Attikon University General Hospital, ads, CIC 604, P. Tsirigotis (52 (52) 44/8)
Athens, Evanghelismos Hospital, ads, CIC 622, I. Baltadakis (61 (63) 46/15)
Athens, Diagnostic & Therapeutic Center 'Hygeia', ads, CIC 643, G. Karianakis (7 (7) 0/7)
Athens, Hellenic Cancer Institute St. Savvas, ads, CIC 751, A. Pouli, E. Sereti, D. Manavoglou (38 (38) 0/38)
Athens, Aghia Sophia Childrens Hospital, peds, CIC 752, V. Kitra-Roussos (49 (70) 31/18)
Athens, Laikon General Hospital, ads, J. Meletis, M. Angelopoulou (no report)
Heraklion, Crete, University Hospital, ads, CIC 435, H. Papadaki, C. Kalpadaki, M. Ximeri (17 (17) 0/17)
Heraklion, Crete, University Hospital Heraklion, peds, CIC 10788, E. Stiakaki (0 (0) 0/0)
Patras, University Hospital of Patras, ads, CIC 281, A. Spyridonidis, M. Liga (37 (40) 25/12)
Piraeus, Metaxa Cancer Hospital, ads, CIC 937, C. Kosmas (4 (8) 0/4)
Thessaloniki, The George Papanicolaou General Hospital, ads, CIC 561, I. Sakellari (132 (133) 63/69)

Hungary: (5 centers: 381 (392) 127/254)
Budapest, Dél-pesti Centrumkórház, National Institute of Hematology, ads, CIC 556, P.Remenyi, L. Gopcsa (189 (192) 78/111)
Budapest, Central Hospital of Southern Pest, peds, CIC 824, G. Kriván (24 (28) 20/4)
Debrecen, University of Debrecen, ads, CIC 648, A. Illes, L. Gergely (104 (108) 22/82)
Miskolc, GYEK, Child Health Centre, peds, CIC 599, R. Simon, A.Kelemen (9 (9) 7/2)
Pécs, University of Pécs, ads, peds, CIC 682, H. Alizadeh, Á. Szomor, S. Kosztolányi, O. Tóth, J. Pammer, Z. Kohl, Z. Szakács (55 (55) 0/55)

Iceland: (1 center: 6 (9) 0/6)
Reykjavik, National University Hospital, ads, CIC 605, I. Hjálmarsdóttir, S. Reykdal (6 (9) 0/6)

Iran: (5 centers: 722 (745) 402/320)
Bandar Abbas, Hormozgan Medical Hospital, ads, CIC 10185, M. Molavi (no report)
Shiraz, Shiraz University of Medical Sciences, Nemazee Hospital, ads, peds, CIC 188, M. Ramzi (187 (197) 75/112)
Tehran, Shariati Hospital, SCT Research Centre, ads, peds, CIC 633, M. Biglari, M. Vaezi, A. Ghavamzadeh, K. Alimoghaddam, M. Yaghmaie (314 (320) 156/158)
Tehran, Childrens Medical Centre, peds, CIC 856, Amir Ali Hamidieh (108 (112) 89/19)
Tehran, Taleghani Hospital Blood and Marrow Transplantation Center, ads, CIC 916, M. Mehdizadeh, A. Hajifathali (65 (65) 40/25)
Tehran, Mofid Children Hospital, peds, CIC 10190, B. Shahin Shamsian (48 (51) 42/6)
Teheran, Mahak Children´s Cancer Hospital, peds, A. Mehrvar (no report)
Urmia, Urmia Medical Sciences University, ads, R. Asghari (no report)

Iraq: (1 center: 61 (61) 24/37)
Sulaimania Kurdistan, HIWA Cancer Hospital, ads, peds, D. Huda, A. Abbas, D. Chra Abdullah, D. Dereen, A. Mohammed, D.T.I. Mahmood, D.C.A. Sabir, D. Vian (61 (61) 24/37)

Ireland: (4 centers: 256 (283) 100/156)
Dublin, St.James Hospital, ads, peds, CIC 257, C. Flynn, P.J. Hayden, L.Bacon (180 (195) 88/92)
Dublin, St. Vincent's Hospital, ads, CIC 541, K. Fadalla (10 (10) 0/10)
Dublin, Our Lady's Hospital of Sick Children, Crumlin, peds, CIC 774, P. Evans (21 (28) 12/9)
Galway, Galway University Hospitals, ads, CIC 408, A. Hayat (45 (50) 0/45)

Israel: (10 centers: 807 (852) 485/322)
Beer Sheba, Soroka University Medical Center, ads, CIC 481, V. Stavi (31 (31) 13/18)
Haifa, Rambam Medical Center, ads, peds, CIC 345, T. Zuckerman (190 (193) 123/67)
Jerusalem, Hadassah University Hospital, ads, peds, CIC 258, P. Stepensky (84 (92) 46/38)
Petach-Tikva, Beilinson Hospital, ads, CIC 409, M. Yeshurun (102 (102) 43/59)
Petach-Tikva, Childrens Medical Center, peds, CIC 755, J. Stein (35 (41) 27/8)
Revohot, Kaplan Hospital, ads, CIC 327, L. Shvidel, K. Filanovsky, Y. Nerklaev, Z. Kopf, E. Yakovenko (7 (7) 0/7)
Tel Aviv, Tel Aviv Sourasky Medical Center, ads, CIC 161, R. Ram (91 (98) 47/44)
Tel Aviv, Dana-Dwek Children's Hospital, Sourasky Medical Centre, peds, CIC 670, R. Elhasid (14 (14) 8/6)

Tel Hashomer, Chaim Sheba Medical Center, peds, CIC 572, A. Toren (66 (72) 52/14)
Tel Hashomer, Sheba Medical Center, ads, CIC 754, A. Nagler, A. Shimoni (187 (202) 126/61)

Italy: (95 centers: 4943 (5522) 1956/2987)
Alessandria, S.S. Antonio e Biagio e C. Arrigo, ads, CIC 825, M. Ladetto, F, Zallio, S. Butera, P. Rivela (60 (63) 36/24)
Ancona, Azienda Ospedale Riuniti di Ancona, ads, peds, CIC 788, A. Olivieri, P. Coccia, G. Mancini (56 (56) 33/23)
Ascoli Piceno, Mazzoni Hospital, ads, CIC 119, P. Galieni (47 (53) 20/27)
Avellino, A.O.S. Guiseppe Moscati, ads, CIC 789, A. Risitano (35 (43) 13/22)
Aviano, CRO IRCCS Aviano, ads, CIC 162, M. Michieli, M. Rupolo, M. Mazzucato (28 (28) 0/28)
Bari, Universitá degli Studi di Bari, ads, CIC 649, P. Carluccio (55 (62) 26/29)
Bari, IRCCS Istituto Tumori "Giovanni Paolo II", ads, CIC 934, A. Guarini (32 (41) 0/32)
Barletta, Hospital of Barletta, ads, CIC 555, G. Tarantini (17 (21) 0/17)
Bergamo, ASST Papa Giovanni XXIII, ads, CIC 658, A. Rambaldi (107 (117) 64/43)
Bologna, San Orsola-Malpighi Hospital, ads, CIC 240, F. Bonifazi (150 (158) 59/91)
Bologna, Instituto Ortopedico Rizzoli, ads, peds, CIC 453, A. Paioli, A. Marrari, M.Pierini, R.Rycther, T.Ibrahim, P.Coluccino (3 (3) 0/3)
Bologna, Policlinico S. Orsola-Malpighi, peds, CIC 790, A. Prete (18 (20) 11/7)
Bolzano, Ospedale San Maurizio, ads, CIC 299, A. Guella, S. Martinelli, T. Aloisi, L. Paoli. D. Dalceggio, S. Cerù, E. Lista, C. Toldo, R. Piffer (21 (28) 0/21)
Bolzano, Hematology Bolzano, Clinical Unit, ads, CIC 2991, I. Cavattoni, A. Kuzina, F. Mosna, N. Pescosta, S. Frisoli (54 (62) 19/35)
Brescia, Azienda Ospedaliera Spedali Civili Di Bresia, ads, CIC 141, D. Russo, V. Radici, G. Magliano (49 (52) 46/3)
Brescia, Azienda Spedali Civili, ads, CIC 288, A. Re, A. Tucci (106 (125) 0/106)
Brescia, Ospedale dei Bambini Spedali Civili, peds, CIC 741, F. Porta, E. Soncini, G. Baresi, S. Rossi, M. Maffeis, G. Graifembergh (15 (16) 13/2)
Brindisi, Perrino Hospital, ads, CIC 920, D. Pastore (36 (39) 20/16)
Busto Arsizio, Ospedale di Circolo di Busto Arsizio, ads, CIC 927, E. Todisco (14 (14) 0/14)
Cagliari, Ospedale per le Microcitemie, peds, CIC 811, A.Piroddi, L.Gaudi Megnetto (9 (9) 6/3)
Cagliari, Binagh Hospital, Armando Businco Centre, ads, peds, CIC 811, E. Piras, G. la Nasa (76 (84) 40/36)
Campobasso, Gemelli Molise Hospital, ads, CIC 1034, A. Bacigalupo (0 (0) 0/0)
Catania, Ospedale Ferrarotto, University of Catania, ads, peds, CIC 792, G. Milone, G. Moschetti, L. Lo Nigro (72 (87) 24/48)
Catania, Ospedale Ferrarotto, University of Catania, ads, CIC 792, Luca Lo Nigro (2 (3) 1/1)
Civitanove Marche, Ospedale di Civitanova Marche, ads, CIC 419, M. Mirabile (13 (13) 0/13)
Como, Valduce Hospital, ads, CIC 473, M. Turrini, A. Gardellini, E. Maino, A. Ripamonti (13 (15) 0/13)
Cremona, U.O. Ematologia CTMO, ads, CIC 226, A. Molteni, P. Spedini, S. Belletti. (7 (7) 0/7)
Cuneo, Azienda Ospedale "S. Croce e Carle", ads, CIC 606, N.Mordini (44 (50) 21/23)
Ferrara, University of Ferrara, ads, CIC 330, A. Cuneo (20 (22) 0/20)
Florence, Azienda Ospedaliera Universitaria di Careggi, ads, CIC 304, R. Saccardi, R. Boncompagni (113 (121) 45/68)
Florence, Azienda Ospedaliero Universitaria Meyer, peds, CIC 1003, V. Tintori (25 (27) 15/10)
Foggia, Azienda Ospedaliero Universitaria, ads, CIC 414, L. Melillo, L. Ciuffreda, R. Cammarota, G. Spinosa, M.P. Sinisi (15 (15) 0/15)
Genova, Ospedale San Martino, ads, CIC 217, E. Angelucci (157 (175) 74/83)
Genova, Istituto Giannina Gaslini, peds, CIC 274, M. Faraci (37 (37) 21/16)
Latina, Ospedale Santa Maria Goretti, ads, CIC 379, E. Ortu la Barbera (32 (37) 0/32)
Lecce, Opsedale Vito Fazzi de Lecce, ads, CIC 868, N. Di Renzo (30 (38) 13/17)
Milan, University of Milan IRCCS, ads, CIC 265, G. Saporiti, F. Cavallaro, S. Girelli, C. Bianchi, M. Bruno Ventre (78 (86) 40/38)
Milan, Ospedale di Niguarda, ads, CIC 294, G. Grillo, C. Vigano (82 (82) 35/47)
Milan, Instituto Europeo di Oncologia, ads, CIC 331, R. Pastano. (41 (41) 14/27)
Milan, Istituto Clinico Humanitas IRCCS, ads, CIC 354, S. Bramanti, B. Sarina (96 (101) 49/47)
Milan, Ist. Nazionale Tumori di Milano, ads, peds, CIC 616, P. Corradini (76 (90) 12/64)
Milan, Istituto Scientifico H.S. Raffaele, ads, peds, CIC 813, F. Ciceri, M. Marcatti (132 (152) 90/42)
Modena, University of Modena, ads, peds, CIC 543, A. Cuoghi, P. Bresciani, M. Luppi, A. Gilioli, A. Messerotti, V. Pioli, G. Palazzi (44 (52) 12/32)

Monza, Ospedale San Gerardo, Universita Di Milano-Bicocca, ads, CIC 544, P. Pioltelli, M. Parma (81 (81) 40/41)
Monza, Ospedale San Gerardo, peds, CIC 279, A. Biondi, A. Balduzzi (26 (30) 25/1)
Napels, AORN Santobono Pausilipon, peds, CIC 341, F.P. Tambaro, F. Cacace, V. Caprioli, M.R. d'Amico, G. de Simone, M.S. Sabbatino, E. Rossitti (24 (25) 18/6)
Napels, AORN Cardarelli Hospital, ads, CIC 607, A. Picardi, M. Celentano, M. Pedata (75 (81) 26/49)
Napels, Frederico ll University, ads, CIC 766, F. Pane, G. Battipaglia (43 (44) 16/27)
Napels, National Cancer Institute IRCCS, ads, CIC 839, G. Marcacci, A. Pinto (50 (63) 0/50)
Novara, Ospedale Maggiore della Carita, ads, CIC 867, G. Gaidano, M. Greco (37 (42) 0/37)
Nuoro, Ospedale San Francesco, ads, CIC 793, A. Palmas, A. Uras (10 (10) 0/10)
Padova, Padua University Hospital, ads, L. Trentin (46 (52) 4/42)
Padova, Clinica di Oncoematologia Pediatrica, peds, CIC 285, A.Biffi, C.Mainardi, M.Tumino, M.Gabelli, M.Pillon, A.Marzollo, L.Cosma, A.V. Strano, A.Paladin (28 (28) 24/4)
Padova, Istituto Oncologia Veneto IOV-IRCCS, ads, CIC 319, A. Brunello, D.Marino (25 (28) 0/25)
Pagani, Hospital A. Tortora, ads, C. Califano (25 (25) 0/25)
Palermo, Ospedale die Bambini, peds, CIC 109, S. Tropia, G. A. Restivo, S. Affronti (1 (2) 0/1)
Palermo, ARNAS Civico Di Cristina, ads, CIC 157, O. Ziino (12 (16) 0/12)
Palermo, A.O.R. Villa Sofia Cervello, ads, peds, CIC 392, L. Castagna, L. Sbriglio (104 (113) 51/53)
Palermo, Ospedale 'La Maddalena', ads, CIC 692, M. Musso, F. Porretto, A. Crescimanno, R. Scalone (110 (136) 17/93)
Parma, University of Palma, ads, CIC 245, L. Prezioso (38 (41) 18/20)
Pavia, IRCCS Policlinico San Matteo, ads, CIC 1006, P. Pedrazzoli (14 (37) 0/14)
Pavia, IRCCS Policlinico San Matteo, ads, CIC 1006, N. Polverelli (96 (96) 77/19)
Pavia, IRCCS Policlinico San Matteo, ads, peds, CIC 1006, M.Zecca, L. Kelly (29 (33) 28/1)
Perugia, Ospedale Santa Maria della Misericordia, ads, peds, CIC 794, A. Carotti (74 (79) 25/49)
Pesaro, AORMN Hospital, ads, CIC 529, G.Visani (28 (37) 12/16)
Pescara, Ospedale Civile, ads, CIC 248, S. Santarone, A. Natale, D. Vaddinelli, R. Spadano (91 (91) 32/59)
Piacenza, Hospital Guglielmo da Saliceto, ads, CIC 163, D.Vallisa (33 (42) 20/13)
Pisa, University of Pisa, ads, peds, CIC 795, G. Casazza, M.Pelosini (70 (72) 29/41)
Potenza, San Carlo Hospital, ads, CIC 861, M. Cimminiello (16 (20) 0/16)
Ravenna, Romagna Metropolitan Transplant Network, ads, CIC 306, F. Lanza (82 (121) 0/82)
Reggio di Calabria, Grande Ospedale Metropolitano, Bianchi Melacrino Morelli, ads, peds, CIC 587, M. Martino, B. Loteta, F. A. Canale (115 (152) 41/74)
Reggio Emilia, Arcispedale S. Maria Nuova, ads, CIC 660, F. Merli, L. Facchini (38 (40) 11/27)
Rionero in Vulture, IRCCS Referral Cancer Center of Basilicata, ads, CIC 185, Dr. Pietrantuono (8 (10) 0/8)
Rome, Università " La Sapienza", ads, CIC 232, A.P. Iori, W. Barberi, S. Capria, S.M. Trisolini, R. Ricci (82 (82) 34/48)
Rome, Ospedale S. Camillo, ads, CIC 287, A. Proia (18 (18) 10/8)
Rome, Università Cattolica S. Cuore, ads, peds, CIC 307, S. Sica, P. Chiusolo, A. Bacigalupo (136 (166) 67/69)
Rome, Rome Transplant Network, ads, CIC 756, R. Cerretti, P. De Fabritiis, L. Rigacci, A. Mengarelli, A. Tafuri, L. Cudillo (200 (210) 48/152)
Rome, IRCCS Ospedale Bambino Gesu, peds, CIC 796, F. Locatelli, F. Galaverna (118 (137) 86/32)
Salerno, AOU San Giovanni di Dio e Ruggi D'Aragona Hospital, ads, CIC 928, C. Selleri, B. Serio, M. Langella, F. D'Alto, V. Giudice (24 (26) 12/12)
San Giovanni Rotondo, Hospital Casa Sollievo Sofferenza, ads, CIC 526, AM. Carella (53 (66) 38/15)
Sassari, Universita Di Sassari, ads, CIC 870, C. Fozza, L. Podda, A. Murtas (11 (11) 0/11)
Siena, Azienda Ospedaliera Universitaria Senese, ads, CIC 321, G. Marotta, M. Tozzi (47 (52) 13/34)
Taranto, Institute of Haematologie, Ospedale Nord, ads, CIC 332, A. Maggi, C. Ingrosso (33 (33) 6/27)
Torino, A.O.U Citta della Salute e della Scienza di Torino, ads, CIC 231, A. Busca, L. Giaccone, I. Dogliotti,C.M. Dellacasa, S. Zompi (116 (116) 45/71)
Torino, University Hospitals Torino, ads, peds, CIC 305, F. Fagioli, E. Vassallo, F. Saglio, F. Carnevale, D. Cilloni, M. De Gobbi (88 (98) 44/44)
Treviso, Presidio Ospedaliero Treviso, ads, CIC 415, F. Gherlinzoni (30 (42) 0/30)
Tricase (Lecce), Hospital C. Panico, ads, CIC 652, V. Pavone (49 (49) 20/29)
Trieste, Istituto per l'Infanzia, IRCCS Burlo Garofolo, peds, CIC 525, N. Maximova, M. Rabusin (8 (10) 6/2)

Trieste, Azienda Sanitaria Universitaria Integrata di Treste, ads, CIC 982, M. Poiani, F. Zaja, G. Desabbata (19 (25) 0/19)
Udine, Azienda Ospedaliero Universitaria di Udine, ads, CIC 705, G. Facchin, F. Patriarca, R. Fanin (99 (104) 54/45)
Varese, Ospedale di Circolo e Fondazione Macchi, ads, CIC 878, A. Ferrario. B. Bianchi. R. Mattarucchi (21 (21) 0/21)
Venice, Ospedale dell'Angelo, ads, CIC 502, C. Skert (58 (70) 31/27)
Verona, Policlinico G. B. Rossi, ads, peds, CIC 623, F. Benedetti, S. Cesaro (78 (78) 38/40)
Viagrande, Istituto Oncologico Del Mediterraneo, ads, CIC 7923, G. Moschetti, F. Galbo, G. MIlone (60 (60) 0/60)
Vicenza, Ospedale S. Bortolo, ads, CIC 797, C. Borghero, F. Elice, A. Lico (49 (56) 18/31)

Jordan: (2 centers: 249 (269) 123/126)
Amman, King Hussein Cancer Centre, ads, CIC 580, A. Tbakhi (175 (194) 67/108)
Amman, Queen Rania Street, Aljubiha, peds, CIC 1058, R. Rihani, M. Flaifel (74 (75) 56/18)
Amman, Istishari Hospital, ads, CIC 487, A. Ahmed Hussein (no report)
Ammann, Arab Medical Center, ads, peds, M. Sarhan (no report)

Kazakhstan: (1 center: 73 (73) 35/38)
Astana, National Research Center for Oncology and Transplantology, ads, V. Kemaikin (73 (73) 35/38)

Latvia: (1 center: 29 (29) 0/29)
Riga, Clinic Linezers, ads, CIC 583, S. Lejiniece, I. Trociukas (29 (29) 0/29)

Lebanon: (2 centers: 244 (252) 117/127)
Beirut, American University of Beirut, ads, peds, CIC 369, A. Bazarbachi (101 (109) 52/49)
Bsalim, Middle East University Hospital, ads, peds, A. Ibrahim (143 (143) 65/78)

Lithuania: (3 centers: 241 (288) 99/142)
Kaunas, University of Health Sciences Kauno Klinikos, ads, CIC 942, R. Gerbutavicius, D. Vaitiekus (58 (67) 17/41)
Vilnius, University Childrens Hospital, peds, CIC 508, J. Rascon, R. Pasaulienė, I Kairienė (24 (29) 16/8)
Vilnius, Santariskiu Klinikos, ads, CIC 644, L. Griskevicius, I. Trociukas, A. Zucenka, L. Kevlicius, V. Jersoviene, G. Buikauskiene, M. Busevici (159 (192) 66/93)

Luxembourg: (1 center: 38 (38) 0/38)
Luxembourg, Center Hospitalier, ads, CIC 1048, S. De Wilde (38 (38) 0/38)

Macedonia: (1 center: 58 (58) 19/39)
Skopje, University Clinic for Haematology, ads, CIC 381, A. Pirkova Veljanovska (58 (58) 19/39)
Skopje, University St. Cyril and Methodius Medical Faculty, ads, CIC 10189, S. Genadieva (no report)

The Netherlands: (14 centers: 1528 (1651) 628/900)
Amsterdam, Academic Med Centre, ads, CIC 247, E.Nur (99 (99) 42/57)
Amsterdam, VU University Medical Center, ads, CIC 588, E. Nur (213 (243) 92/121)
Amsterdam, Antoni Van Leeuwenhoek Hospital, ads, CIC 976, S. C. Linn (6 (6) 0/6)
Enschede, Medisch Spectrum Twente, ads, CIC 360, M.R. Schaafsma, MC. Legdeur (40 (47) 0/40)
Groningen, University Medical Centre UMCG, ads, CIC 546, A. Mäkelburg, G. Huls, C.L.E. Hazenberg, G. Choi, E. Bremer, M. Bellido, W. Plattel, L.M. Morsink, T (152 (152) 70/82)
Leiden, University Hospital, ads, peds, CIC 203, J.H. Veelken (192 (207) 147/45)
Maastricht, University Hospital, ads, CIC 565, G. Van Gorkom (110 (126) 40/70)
Nieuwegein, St. Antonius Hospital, ads, CIC 200, H.R. Koene, A. Reitsma, K. Molenkamp (43 (47) 0/43)
Nijmegen, University Hospital, ads, CIC 237, N. Schaap, M. Roeven (123 (129) 38/85)
Rotterdam, Erasmus MC Cancer Institute, ads, CIC 246, A.E.C. Broers (238 (257) 96/142)
The Hague, Haga Hospital Leyenburg, ads, CIC 547, T. Netelenbos, J.L. Kerkhoffs, L. Bohmer, P. Ypma, D.v. Lammeren,S. Kersting, (52 (57) 0/52)
Utrecht, University Medical Centre UMCU, ads, CIC 239, J. Kuball, M. de Witte (143 (144) 62/81)
Utrecht, Princess Maxima Centre for Ped Oncology, peds, CIC 352, M. Bierings (75 (91) 41/34)
Zwolle, Isala Klinieken, ads, CIC 548, G.L. van Sluis (42 (46) 0/42)

Nigeria: (1 center: 4 (4) 3/1)
Benin, University Hospital Benin City, ads, peds, N. Bazuaye (4 (4) 3/1)

Norway: (5 centers: 404 (476) 176/229)
Bergen, Helse Bergen, University Hospital, ads, CIC 197, A. Ahmed (68 (81) 22/46)
Oslo, The Norwegian Radium Hospital, ads, J. Riise, M. Rodrigues, S. Kvaloy (43 (43) 0/43)
Oslo, Oslo University Hospital, ads, peds, CIC 235, T. Gedde-Dahl, J. Büchner (239 (285) 154/85)
Tromso, University Hospital North Norway, ads, A. Vik, G. Knutsen (14 (16) 0/14)
Trondheim, St. Olavs Hospital, ads, O. Hjertner (40 (51) 0/40)

Poland: (20 centers: 1635 (1754) 727/908)
Bydgoszcz, Nicolaus Copernicus University, peds, CIC 764, J. Styczynski, R. Debski (25 (28) 17/8)
Cracow, Jagiellonian University CMUJ, ads, T. Sacha (102 (102) 43/59)
Cracow, University Children's Hospital JUMC, peds, CIC 507, J. Gozdzik (21 (24) 13/8)
Gdansk, Medical University, ads, CIC 799, J. Maciej Zaucha, M. Bieniaszewska, A. Piekarska, M. Dutka, D. Zielińska, W. Prejzner, P. Sobieralsk (131 (131) 58/73)
Gliwice, Maria Curie Memorial Cancer Centre, ads, CIC 428, S. Giebel (194 (228) 92/102)
Katowice, Silesian Medical Academy, ads, CIC 677, G. Helbig (281 (311) 150/131)
Lodz, Medical University of Lodz, ads, CIC 171, T. Robak (82 (82) 15/67)
Lublin, Childrens University Hospital, peds, CIC 678, K. Drabko, J. Kowalczyk (9 (9) 7/2)
Lublin, University Medical School, ads, CIC 695, T. Gromek, M. Wach, A. Walter-Croneck, W. Legiec (35 (42) 2/33)
Poznan, Poznan University of Medical Sciences, ads, CIC 730, L. Gil, A. Agnieszka (119 (119) 60/59)
Poznan, University of Medical Sciences, Pediatric Hematology, peds, CIC 641, J. Wachowiak, K. Derwich, A. Sobkowiak-Soboerajska, A. Pieczonka, M. Deręgowski, M. Barańska, A. Gał (13 (13) 10/3)
Szczecin, Uniwersytecki Szpital Kliniczny, ads, S. Milczarek, B. Baumert, K. Sommerfeld, E. Borowiecka, B. Osekowska, Ed (66 (67) 27/39)
Warsaw, Institue of Haematology and Blood Transfusion, ads, CIC 693, K. Hałaburda, B. Nasiłowska-Adamska, M. Bajorek-Muzyczka, J. Szypnicki (81 (93) 46/35)
Warsaw, Maria Sklodowska Curie National Research Institute, ads, CIC 800, J. Romejko-Jarosinska, L.Targonski, J.Walewski (70 (77) 0/70)
Warsaw, Military Institute of Health Services, ads, CIC 816, P. Rzepecki, K. Sulek (53 (53) 11/42)
Warsaw, Central Clinical Hospital, ads, CIC 954, G. Basak, P. Rusicka, W. Wiktor-Jedrzejczak, P. Boguradzki (82 (88) 37/45)
Warsaw, Oncology Clinic, peds, CIC 1049, I. Malinowska, M. Romiszewski (20 (20) 9/11)
Wroclaw, Lower Silesian Center, ads, CIC 538, A. Lange (55 (64) 29/26)
Wroclaw, University Hospital SPSK 1, ads, CIC 699, T. Wrobel (143 (144) 56/87)
Wroclaw, Cape of Hope Medical University, peds, CIC 817, K. Kalwak, J. Owoc-Lempach, M. Ussowicz, M. Mielcarek-Siedziuk, J. Fraczkiewicz, I.Olejnik, I.Dachow (53 (59) 45/8)

Portugal: (6 centers: 556 (578) 146/410)
Coimbra, University Hospital, ads, CIC 905, C. Geraldes, A. Roque, Ana Bela Sarmento (50 (50) 0/50)
Lisbon, Instituto Portugues de Oncologia, ads, peds, CIC 300, M. Nuno (108 (113) 51/57)
Lisbon, Hospital de Santa Maria, ads, CIC 636, J. F. Lacerda, F. Lourenço, C. Martins, A. Rodrigues, A. Garção (81 (81) 30/51)
Lisbon, H. St. Antonio dos Capuchos, ads, CIC 826, F. Costa, G. Ferreira, R. Gerivaz, A. Tome, C. Damiao, F. Pereira, P. Lage (59 (63) 0/59)
Porto, Instituto Portugues de Oncologia, ads, peds, CIC 291, C.P. Vaz, H. SIlva (143 (151) 53/90)
Porto, Hospital St. Joao, ads, CIC 329, F. Trigo, R. Pinto (115 (120) 12/103)

Romania: (5 centers: 259 (271) 97/162)
Bucharest, Fundeni Clinical Institute, ads, CIC 427, A. Tanase (161 (169) 65/96)
Bucharest, Coltea Clinical Hospital, ads, CIC 912, A. Colita, C. Ghimici, R. Manolache, N. Berbec, L. Neacsu, C. Stoica, F. Gradinaru (21 (21) 3/18)
Bucharest, Fundeni Clinical Centre, peds, CIC 935, A. Colita, C. Jercan, A. Bica, A. Marcu, I. Avramescu (31 (35) 27/4)
Targu-Mures, Sectia Clinica de Hematologie si Transplant Medular, ads, E. Lazar, J.B. Kopeczi, E. Kakucs (27 (27) 1/26)
Timisoara, Louis Turcanu Hospital, ads, peds, CIC 174, S. Arghirescu, A. Isac, C. Jinca, A. Pascalau, A. Oprisoni, M. Serban (19 (19) 1/18)

Russia: (14 centers: 1507 (1604) 660/847)
Ekaterinburg, Regional Hospital No. 1, ads, T.S. Konstantinova, V.A. Shalaev (57 (57) 25/32)
Ekaterinburg, Royal Children's Hospital, peds, L. Fechina (44 (45) 37/7)
Moscow, Cancer Research Center N. N. Blokhin, ads, G. Petrova, N. Nikiforova, I. Monin, A. Efirkina, E. Orlova, L. Maltzeva (63 (64) 9/54)
Moscow, Main Military Clinical Hospital, ads, O.A. Rukavitsyn, V. Pop (14 (15) 0/14)
Moscow, Central Clinical Hospital (CCHPA), ads, S. Shamansky (9 (9) 0/9)
Moscow, National Pirogov Medical Centre, ads, V. Melnichenko, N. Mochkin (274 (274) 6/268)
Moscow, The Russian Children's Research Hospital, peds, CIC 411, E. Skorobogatova (88 (96) 74/14)
Moscow, Federal Research Center for Pediatric Hematology, peds, CIC 694, A. Maschan, D. Balachov (153 (181) 110/43)
Moscow, N.N. Blokhin National Medical Research Center, peds, CIC 893, K. Kirgizov (184 (184) 83/101)
Moscow, Research Haematology Center of RAS, ads, CIC 930, E. Parovichnikova (no report)
Moscow, FMBC Burnasyan, ads, A. Davtyan, A.E. Baranov (no report)
Novosibirsk, Institute of Clinical Immunolgy, ads, V. Sergeevuicheva (47 (47) 5/42)
Samara, Samara Kalinin Regional Hospital, ads, V.A. Rossiev (10 (10) 0/10)
St. Petersburg, Russian Scientific and Research Institute of Haematology, ads, S. Voloshin (58 (59) 9/49)
St. Petersburg, Memorial Petrov National Medical Research Cancer Center, ads, peds, I.Zyuzgin, I.Ishmatova, A.Zverkova, S.Volchenkov, N.Kolupaev, S.Elkhova, Y.Nikulina, L.Kramynin, E.D (118 (118) 28/90)
St. Petersburg, First State Pavlov Medical University of St. Petersburg, ads, peds, CIC 725, A. Kulagin, L. Zubarovskaya, I. Moiseev (388 (445) 274/114)
St. Petersburg, Federal Centre V.A. Almazov, ads, A. Zaritskey, D. Motorin (no report)

Saudi Arabia: (5 centers: 762 (822) 558/204)
Dammam, King Fahad Specialist Hospital, ads, peds, CIC 441, H. Al-Hashmi (no report)
Jeddah, King Faisal Hospital, ads, peds, CIC 858, M. Bayoumi (102 (113) 64/38)
Riyadh, King Fahad Medical City, ads, peds, CIC 159, M. Al-Harbi (107 (122) 66/41)
Riyadh, King Faisal Specialist Hospital, ads, peds, CIC 397, M. Al Jurf (224 (239) 150/74)
Riyadh, King Faisal Specialist Hospital, peds, CIC 981, A. Al Ahmari, S. Jafri, M. S. Zulfiqar (133 (151) 120/13)
Riyadh, King Abdul Aziz Medical City, ads, peds, CIC 444, M. Al Zahrani (196 (197) 158/38)
Riyadh, Prince Sultan Military Medical City, ads, CIC 818, S. Al Otaibi (no report)

Serbia: (4 centers: 221 (225) 71/150
Belgrade, Mother and Child Health Institute of Serbia "Dr Vukan Čupić", peds, CIC 358, D. Vujić, M. Simić, B. Gobeljić, E. Lazić, A. Borojević, B. Eremija (26 (26) 17/9)
Belgrade, Clinical Center of Serbia, ads, CIC 373, M. Todorovic Balint (102 (103) 26/76)
Belgrade, Military Medical Academy, ads, CIC 582, D. Stamatovic (72 (75) 21/51)
Novi Sad, Clinical Center of Vojvodina, ads, CIC 655, A. Savic (21 (21) 7/14)

Slovakia: (5 centers: 222 (247) 77/145)
Banská Bystrica, Roosevelt Hospital, ads, CIC 333, E. Kralikova, J. Holasova, A. Pucatova (19 (20) 0/19)
Bratislava, National Cancer Institute, ads, CIC 368, A. Vranovsky (67 (77) 9/58)
Bratislava, University Hospital, ads, CIC 610, L. Sopko (85 (94) 52/33)
Bratislava, University Hospital, peds, CIC 684, P. Svec, I. Bodova, T. Sykora, J. Adamcakova, M. Fussiova (24 (29) 16/8)
Kosice, University Hospital, ads, T. Guman, N. Stecova (27 (27) 0/27)

Slovenia: (1 center: 115 (136) 42/73)
Ljublijana, University Medical Centre, ads, peds, CIC 640, M. Sever, S. Avčin (115 (136) 42/73)

South Africa: (10 centers: 386 (388) 128/258)
Cape Town, Constantiaberg Medical Clinic, ads, J.M.G. du Toit, P. Loebenberg, D. Kotze, L. Sassman (40 (40) 12/28)
Cape Town, Melomed Hospital Tokai, ads, S. Nahrwar (5 (5) 1/4)
Cape Town, Netcare Kuils River Hospital, ads, peds, H. Koornhof (71 (72) 26/45)

Cape Town, Groote Schuur Hospital, ads, peds, CIC 512, E. Verburgh (56 (56) 28/28)
Cape Town, UCT Private Academic Hospital, ads, peds, N. Novitzky (no report)
Durban, Inkosi Albert Luthuli Hospital, ads, peds, S. Parasnath (16 (16) 5/11)
Durban, Capital Haematology Hospital, ads, peds, J.P.Singh (18 (19) 3/15)
Johannesburg, Chris Hani Baragwanath Hospital, ads, V. Philip, A. Lakha, F. Waja, G. Laudin, L. Goqwana, R. Jassat (23 (23) 1/22)
Johannesburg, Wits Donald Gordon Medical centre, ads, peds, CIC 483, J. Thomson (37 (37) 15/22)
Pretoria, Netcare Pretoria East Hospital, ads, CIC 456, D. Brittain, A. McDonald (93 (93) 29/64)
Randburg, Netcare Olivedale Hospital, ads, CIC 889, K. Gunther. D. Brittain (27 (27) 8/19)

Spain: (72 centers: 3464 (3651) 1398/2066)
Alicante, Hospital General, ads, P. Fernandez Albellan (40 (40) 0/40)
Almeria, Hospital Universitario Torrecardenas, ads, CIC 486, M.J. Garcia Perez (16 (16) 0/16)
Barakaldo Vizcaya, Hospital de Cruces, ads, peds, CIC 393, J. Garcia-Ruiz, J. Mateos-Mazon (67 (78) 0/67)
Barcelona, Hospital Mutua de Terrassa, ads, J. M. Marti Tutusaus (35 (35) 0/35)
Barcelona, Hospital Clinic, ads, CIC 214, M. Rovira (100 (107) 54/46)
Barcelona, Santa Creu i San Pau, peds, CIC 260, I. López Torija (8 (8) 8/0)
Barcelona, Santa Creu i Sant Pau, ads, peds, CIC 260, R. Martino, A. Esquirol, I. López (112 (115) 64/48)
Barcelona, Hospital M. Infantil, Vall d'Hebron, peds, CIC 422, C. Diaz de Heredia (32 (39) 23/9)
Barcelona, Hospital General Vall d'Hebron, ads, CIC 584, D. Valcarcel (52 (54) 27/25)
Barcelona, Hospital Germans Trias i Pujol, ads, CIC 613, A. Torrent (61 (62) 28/33)
Barcelona, Hospital Sant Joan de Deu, peds, CIC 668, M. Trabazo, A. Alonso, I. Badell-Serra (37 (40) 31/6)
Barcelona, Institute Catala d'Oncologia, Hospital Duran i Reynals, ads, CIC 759, A. Mussetti, A. Sureda (99 (99) 35/64)
Caceres, Hospital San Pedro de Alcantara, ads, S. Cáceres, F. Ibañez, V. Higero, J.M. Bergua (44 (44) 0/44)
Cadiz, University Hospital of Jerez de la Frontera, ads, S. Garzon-Lopez, R. Saldaña Moreno, B. López Pulido, E. Gálvez de la Villa, L. Domínguez Acosta, A. (56 (56) 23/33)
Castellon de La Plana, Hospital General de Castellon, ads, R. Garcia-Boyero (20 (22) 0/20)
Cordoba, Hospital Reina Sofia, ads, peds, CIC 238, C. Herrere Arroyo, C. Martin Calvo, V. Martin Palanco (76 (76) 38/38)
Galdakao, Hospital de Galdakao, ads, T.Carrascosa, E.Gainza, I.Etxeguren (12 (12) 0/12)
Girona, Institut Catala d'Oncologia, Josep Trueta, ads, CIC 433, D. Gallardo (34 (38) 0/34)
Granada, Hospital Virgen de la Nieves, ads, CIC 559, M. Jurado Chacon (78 (78) 30/48)
Jaen, Hospital Cuidad de Jaen, ads, F. Almagro Torres (16 (16) 0/16)
La Coruna, Complejo Hospitalario de A Coruna, ads, CIC 361, J P. Torres Carrete, M R. Varela Gomez (60 (64) 21/39)
La Laguna, Tenerife, University Hospital Canary Isles, ads, M.T. Hernandez-Garcia (37 (37) 0/37)
Las Palmas Canary Isles, Hospital Insular, ads, J. Gonzalez-San Miguel (26 (26) 0/26)
Las Palmas Canary Isles, Hospital de Gran Canaria 'Dr. Negrin', ads, CIC 537, M.del Mar Perera, A.Suarez, H.Luzardo (86 (87) 51/35)
Leon, Hospital Universitario de Leon, ads, CIC 426, N de Las Heras (21 (23) 0/21)
Lleida, Hospital Arnau de Vilanova, ads, CIC 885, A. Garcia Guinon (10 (10) 0/10)
Logrono, Hospital San Pedro, La Rioja, ads, M. Najera Irazu, M. Hermosilla (14 (14) 0/14)
Lugo, Hospital Lucus Augusti, ads, E. Lavilla (24 (31) 0/24)
Madrid, Hospital Universitario San Carlos, ads, M.Paz Martin, C. Benavente (11 (11) 0/11)
Madrid, Hospital Universitario Quiron Salud/Hospital Moncloa, ads, JM. Fernadez-Ranada, A. Escudero, M. Chamarro (45 (45) 5/40)
Madrid, Hospital Principe Asturias, Alcala de Henares, ads, Dr. Lopez Rubio, E. Magro Mazo (14 (14) 0/14)
Madrid, Hospital Universitario de Getafe, ads, N. Somolinos, C. Escolano,I. Delgado (9 (9) 0/9)
Madrid, Hospital Universitario Sanchinarro, ads, peds, J. Pérez de Oteyza (14 (14) 1/13)
Madrid, Hospital Severo Ochoa, Leganés, ads, P. Sanchez Godoy (5 (5) 0/5)
Madrid, Hospital de la Princesa, ads, CIC 236, A. Alegre, A. Figuera, B. Aguado, R. de la Cámara, V. Gómez, C. Cámara, C. Jiménez (42 (42) 27/15)
Madrid, Fundacion Jimenez Diaz, ads, CIC 309, JL. Lopez-Lorenzo (59 (65) 26/33)

Madrid, Hospital Doce de Octubre, ads, CIC 382, J. Martinez Lopez, M. Calbacho, E. Gil Sanchez (109 (109) 30/79)
Madrid, Hospital Univeristario Materno Infantil Gregorio Maranon, peds, CIC 410, C. Belendez (14 (16) 10/4)
Madrid, Hospital Ramon y Cajal, ads, CIC 615, J. Lopez-Jimémez (100 (101) 45/55)
Madrid, Hospital Universitario Puerta de Hierro, ads, CIC 728, R. Duarte, I Salcedo (57 (57) 35/22)
Madrid, Hospital Niño Jesus, peds, CIC 732, M. González, B. Molina, M. Barrilero, B. Segura, J. Ruiz (34 (40) 27/7)
Madrid, Hospital Universitario La Paz, ads, peds, CIC 734, A. Perez-Martinez, R. De Paz (77 (81) 45/32)
Madrid, Hospital Universitario Sanitas La Zarzuela, ads, CIC 779, R. De la Camara (2 (2) 0/2)
Madrid, Hospital General Universitario Gregorio Maranon, ads, CIC 819, M. Kwon, C. Muñoz, S. Sabell (70 (76) 48/22)
Malaga, Hospital Virgen de la Victoria, ads, CIC 476, A. Rosell Mas (26 (26) 0/26)
Malaga, Hospital Regional Malaga, ads, peds, CIC 576, M-J. Pascual-Cason (117 (134) 60/57)
Murcia, Hospital Virgen de la Arrixaca, ads, peds, CIC 323, JM. Moraleda, A. Sanchez-Salinas (75 (85) 41/34)
Murcia, Hospital General Universitario Morales Meseguer, ads, CIC 735, I. Heras, M. Luisa Lozano (42 (45) 11/31)
Orense, Complejo Hospital Cristal-Pinor, ads, J-L. Sastre-Moral (11 (11) 0/11)
Oviedo, Hospital Covadonga, Central Asturias, ads, peds, CIC 642, S. Gonzalez-Müniz (97 (98) 36/61)
Palma de Mallorca, Hospital son Llatzer, ads, CIC 110, J. Bargay-Lleonart (18 (20) 0/18)
Palma de Mallorca, Hospital Uni. Son Espases, ads, peds, CIC 722, A. Sampol (46 (50) 16/30)
Pamplona, Hospital de Navarra, ads, CIC 577, T. Zudaire (43 (48) 23/20)
Pamplona, Clinica Universitaria de Navarra, ads, peds, CIC 737, J. Rifon (52 (54) 35/17)
Pontevedra, Hospital Montecelo, ads, A-M. Dios Loureiro (24 (24) 0/24)
Salamanca, Hospital Clinico, ads, peds, CIC 727, L.L. Corral (148 (160) 77/71)
San Sebastian, Hospital Universitario Donostia, ads, peds, CIC 598, C.M.P. Santos, J. J. Ferreiro Martinez, M. de la Cruz Viguria Alegria, D. Bourantas (99 (113) 57/42)
Santander, Hospital Universitario Marqués de Valdecilla, ads, CIC 242, M. Colorado Araujo (57 (65) 35/22)
Santiago de Compostela, Hospital Clinico Universitario, ads, peds, CIC 570, J.L. Bello Lopez (50 (50) 25/25)
Sevilla, Hospital Universitario Virgen del Rocio, ads, peds, CIC 769, J. A. Pérez-Simón (156 (156) 79/77)
Tarragona, Hospital Joan XXIII de Tarragona, ads, CIC 896, C. Talarn Forcadell (29 (32) 0/29)
Tenerife Canary Isles, Hospital N. S. De la Candelaria, ads, P. Rios Rull, A. Figueros, L.F. Avila (25 (28) 0/25)
Valencia, Instituto Valenciano de Oncologia, ads, A. Avaria, C. Salazar (7 (7) 0/7)
Valencia, Hospital Arnau de Vilanova de Valencia, ads, A. Lopez Martinez (11 (11) 0/11)
Valencia, Hospital Doctor Peset, ads, P. Ribas-Garcia, A. Garcia Feria (15 (19) 0/15)
Valencia, Hospital Clinico de Valencia, ads, peds, CIC 282, C. Solano (70 (74) 33/37)
Valencia, Hospital Universitario La Fe, ads, CIC 663, J. Sanz, G.F.Sanz (126 (133) 77/49)
Valencia, Hospital Universitario La Fe, peds, CIC 653, J.M. Fernandez Navarro (21 (23) 14/7)
Valladolid, Hospital Rio Hortega, ads, CIC 611, A. Cantalapiedra Díez, E. Bonis Izquierdo, B. González Mena, M.C. González Gómez, V. Molperes Sanz, (39 (39) 8/31)
Vigo, CHUVI Hospital Alvaro Cunqueiro, ads, CIC 421, C. Albo Lopez (65 (72) 25/40)
Zaragoza, Clinico Universitario Lozano Blesa, ads, L. Palomera Bernal (16 (16) 0/16)
Zaragoza, Hospital Miguel Servet, ads, CIC 1037, I. Izquierdo, B. de Rueda, A. Aules, I. Murillo, P. Delgado (44 (44) 14/30)

Sweden: (7 centers: 698 (752) 307/391)
Goteborg, CHECT Sahlgrenska University Hospital, ads, peds, CIC 289, J. Johansson, K. Mellgren (127 (140) 44/83)
Linköping, University Hospital, ads, CIC 740, A. Bergendahl Sandstedt (27 (27) 27/0)
Lund, University Hospital, ads, peds, CIC 283, S. Lenhoff, J. Toporski (147 (162) 72/75)
Örebro, Medical Center Hospital, ads, CIC 738, P. Kozlowski (25 (27) 0/25)
Stockholm, Karolinska University Hospital, ads, peds, CIC 212, S. Mielke (193 (195) 96/97)
Umea, Umea University Hospital, ads, CIC 731, C. Isaksson (64 (71) 22/42)
Uppsala, University Hospital, ads, peds, CIC 266, K. Carlson, N. Jackmann (115 (130) 46/69)

Switzerland: (10 centers: 691 (785) 306/385)

Aarau, Kantonsspital Aarau, ads, peds, CIC 316, S. Gerull (44 (49) 0/44)
Basel, Universitätsspital Basel, ads, peds, CIC 202, J. Passweg, D. Heim, J. Halter (154 (172) 124/30)
Bellinzona, Ospedale San Giovanni, ads, CIC 829, G. Stüssi, M. Baglioni, L. Orlando, B. Loconte, E. Lerch, A. Stasia, F. Bergamini, R. Bordone, E. Sc (8 (12) 0/8)
Bern, Inselspital, ads, peds, CIC 221, T. Pabst, M. Daskalakis (109 (125) 0/109)
Geneva, Hôpital Cantonal Universitaire, ads, peds, CIC 261, Y. Chalandon, M. Ansari (70 (74) 70/0)
Lausanne, CHUV, ads, CIC 820, M. Duchosal (63 (81) 0/63)
St. Gallen, Kantonsspital, ads, CIC 324, F. Hitz (27 (30) 0/27)
Zurich, University Hospital, ads, peds, CIC 208, D. Schneidawind, T. Benoit (148 (171) 84/64)
Zurich, Universitäts Kinderklinik, peds, CIC 334, T. Güngör (32 (34) 28/4)
Zurich, Hospital Hirslanden, ads, CIC 638, Ch. Renner (36 (37) 0/36)

Syria: (1 center: no report)
Damascus, Tishreen Hospital, ads, peds, CIC 10853, De. S. Elias Soulaiman (no report)

Tunisia: (1 center: 69 (72) 28/41)
Tunis, National BMT Centre, ads, peds, CIC 183, N. Ben Abdeljelil (69 (72) 28/41)

Turkey: (59 centers: 3216 (3322 (1711/1505)
Adana, Balcali Hospital, Cukurova University, ads, B. Güvenc (34 (34) 11/23)
Adana, Cukurova University Balcali Hospital, ads, S. Gultekin (4 (4) 1/3)
Adana, Adana Acibadem Hospital, peds, CIC 454, A. Antmen (46 (46) 46/0)
Adana, Baskent University Adana, ads, peds, CIC 589, H. Ozdogu, C. Boga, S. Asma (81 (87) 49/32)
Ankara, Memorial Hospital Ankara, ads, peds, F. Avcu (73 (82) 43/30)
Ankara, Ankara Baskent Hospital, ads, E. Koca (6 (6) 0/6)
Ankara, Lösante Hospital, peds, A.Emin Kurekci (51 (51) 51/0)
Ankara, SBU Gülhane Training and Research Hospital, ads, M. Barış Aykan (103 (103) 18/85)
Ankara, Ankara Bilkent City Hospital Pediatric Clinic, peds, N. Özbek (76 (76) 71/5)
Ankara, Private Medicana International Hospital, ads, U. Sahin (132 (134) 75/57)
Ankara, Hacettepe University Medical School, Sihhiye, ads, CIC 168, H. Goker, H. Demiroglu (50 (50) 29/21)
Ankara, Gazi University Medical School, Besevler, ads, CIC 169, Z.N. Ozkurt, Ö. Karacaoglu (71 (78) 40/31)
Ankara, Gazi University Medical School, Besevler, peds, CIC 182, U. Kocak (13 (13) 13/0)
Ankara, Hacettepe Ihsan Dogramaci Childrens Hospital, peds, CIC 399, F. Visal Okur (13 (13) 13/0)
Ankara, Ankara Bayindir Hospital, ads, CIC 412, A.Ugur Ural, F. Arpaci, S. Karaoglanoglu Akbas (8 (8) 3/5)
Ankara, Ankara University Faculty of Medicine, Dikimevi, ads, CIC 617, P. Topguoglu, K. Meltem (83 (89) 31/52)
Ankara, University of Ankara, Cebeci, peds, CIC 620, T. Lleri, E. Unal (20 (21) 19/1)
Ankara, Numune Education and Research Hospital, ads, G. Özet (no report)
Ankara, Dr. A. Yurtaslan Oncology Training and Research Hospital, ads, F. Altuntas, M. Sinan Dal (no report)
Ankara, Liv Hospital, ads, O. Nevruz (no report)
Ankara, Lösant Hospital, ads, Z. Gokgoz (no report)
Ankara, Ozel Koru Hospital, ads, CIC 1046, A. Ugur Bilgin (no report)
Ankara, Yildirim Bayazit Training and Research Hospital, ads, M. Albayrak (no report)
Antalya, Antalya Education and Research Hospital, ads, I. Nizam Özen (15 (15) 1/14)
Antalya, Akdeniz University School of Medicine, ads, CIC 685, O.K. Yucel, O. Salim , S.C. Kazan , M. Uslu (62 (63) 36/26)
Antalya, Akdeniz University Medical School, peds, CIC 618, A. Kupesi (24 (24) 19/5)
Antalya, Medstar Antalya Hospital, Cakirlar, ads, CIC 864, M. Çetin, M. Özdoğan, R. Saba, B. Deveci (102 (102) 48/54)
Antalya, Medical Park Antalya Hospital, Lara, peds, CIC 911, A. Yesilipek (85 (97) 85/0)
Atakum, Özel Sansung Medicalpark Hospital, peds, H.E. Ozyurek, E. Sahin (no report)
Aydin, Adnan Menderes University Medical Faculty, ads, CIC 187, Z. Bolaman, I. Yavasoglu, A. Turgutkaya, O. Asici (41 (41) 21/20)
Bursa, Uludag University School of Medicine, ads, V. Özkocaman (no report)
Bursa, Uludag University School of Medicine, peds, AM. Günes, M. Evim (12 (12) 7/5)
Denizli, Pamukkale University Hospital, ads, S. Kabukcu (50 (50) 50/0)

Diyarbakir, Dicle University Faculty, ads, O. Ayyildiz, A. Karakus (no report)
Erzurum, Atatürk University, ads, G. Sincan (9 (9) 2/7)
Eskisehir, ESOGÜ Pediatric Hospital, peds, Z.C. Özdemir, Ö. Bör, D. İşyapar (8 (8) 7/1)
Eskisehir, Osmangazi University, ads, E. Gündüz (no report)
Istanbul, Medicalpark Bahçelievler Hospital, ads, A. Gülsan Sucak (77 (91) 36/41)
Istanbul, Yeditepe Universtity Hospital, peds, N. Yalman, C. Timur (4 (4) 3/1)
Istanbul, Yeditepe University Hospital, ads, CIC 416, A. Özkan (no report)
Istanbul, Hisar Intercontinental Hospital, ads, A.Eser, E.Büyük (64 (64) 23/41)
Istanbul, Sisli Memorial Hospital, peds, A. Tanyeli (26 (26) 21/5)
Istanbul, Medical Park Göztepe, ads, C. Adigüzel (no report)
Istanbul, Medical Park Goztepe, peds, G. Karasu, SC. Kilic (95 (103) 85/10)
Istanbul, Acidadem Atakent Hospital, ads, S. Sami Karti, A. Uzay (158 (158) 55/103)
Istanbul, Bahcelievler Memorial Hospital HSCTU Center, ads, E. Tekgündüz (122 (126) 39/83)
Istanbul, Emsey Hospital, ads, CIC 355, S. Omay, Y. Ünsal (118 (118) 52/66)
Istanbul, Istanbul Medipol University, ads, CIC 445, L. Gül Kaynar (143 (143) 64/79)
Istanbul, Istanbul Medipol University, peds, CIC 446, Y. Yaman, S. Anak (27 (27) 20/7)
Istanbul, Bahcelievler Medical Park Hospital, ads, G. Sucak, G. Özgür (no report)
Istanbul, Medical Park Bahcelievler Hospital, peds, CIC 448, T. Fisgin, C. Bozkurt (75 (91) 65/10)
Istanbul, Acibadem University Altunizade Hospital, ads, CIC 468, S. Ratip, E. Ovali (51 (52) 31/20)
Istanbul, Acibadem University Altunizade Hospital, peds, CIC 457, G. Öztürk (18 (20) 15/3)
Istanbul, Yeniyuzyil University, Gaziosmanpasa Hospital, ads, CIC 475, H. Goksoy (no report)
Istanbul, Yeniyuzyil University, Gaziosmanpasa Hospital, peds, CIC 459, B. Malbora (no report)
Istanbul, Marmara University Hospital, ads, CIC 714, T. Firatli-Tuglular, T. Toptas (30 (30) 17/13)
Istanbul, University of Istanbul, ads, CIC 760, I. Yonal-Hindilerden, M. Aktan (44 (48) 15/29)
Istanbul, Cerrahpasa Medical Faculty, ads, CIC 761, M.Cem Ar, T.Elverdi, F.Uzun (15 (16) 3/12)
Istanbul, Medicana International Hospital, ads, CIC 919, Y. Koc (51 (55) 27/24)
Istanbul, Koç University Hospital, ads, CIC 943, Ü. Üre (91 (91) 27/64)
Istanbul, Florence Nightingale Sisli Hospital, ads, CIC 994, M. Arat (109 (109) 63/46)
Istanbul, Kolan International Hospital, ads, CIC 914, E. Kurtoglu (no report)
Istanbul, Medical Park Hospitals, ads, B. Eser (no report)
Istanbul, Sisli Memorial Hospital, ads, S. Izmir Güner (no report)
Izmir, Tepecik Research and Educational Hospital, peds, H. Öniz (1 (1) 0/1)
Izmir, Ege University Medical Faculty, Bornova, peds, CIC 621, S. Aksoylar (29 (32) 25/4)
Izmir, Ege University Medical Faculty, Bornova, ads, CIC 628, F. Vural, G. Saydam, N. Soyer, A. Güneş, B. Vatansever (76 (78) 34/42)
Izmir, Dokuz Eylul University, ads, CIC 688, G. H. Özsan. H. Ören, S. Namdaroğlu (77 (77) 17/60)
Izmir, Medicalpark Private Hospital, Karsiyaka, ads, S.Cagirgan, Kahraman, C. Acarlar (no report)
Izmir, Izmir Bozyaka Hospital, ads, O. Bilgir, D. Cekdemir (no report)
Kayseri, Erciyes University Hospital, ads, CIC 627, A. Unal, M. Keklik (60 (60) 19/41)
Kayseri, Erciyes University Faculty of Medicine, peds, CIC 913, M. Karakukcu (55 (58) 46/9)
Kocaeli, Kocaeli University Hospital, ads, P. Tarkun (32 (32) 2/30)
Kocaeli, Kocaeli University Hospital, ads, E. Zengin (2 (2) 2/0)
Kocaeli, Anadolu Medical Center Hospital, ads, CIC 440, Z. Gülbas (81 (81) 34/47)
Konya, Necmettin Erbakan, Meram University Medical Hospital, ads, Ö. Ceneli (25 (25) 0/25)
Malatya, Inonu University Targut Özal Medical Centre, ads, M. Ali Erkurt, E. Kaya (164 (164) 65/99)
Malatya, Inönu University Hospital, peds, A. Akyay (10 (10) 8/2)
Manisa, Celal Bayer University, ads, I. Aydogdu (no report)
Samsun, Ondokuz Mayis University, peds, C. Albayrak (14 (14) 9/5)
Trabzon, KTU Farabi Hospital, ads, E. Nas, M. Sonmez (no report)

Ukraine: (7 centers: 320 (335) 89/231)
Cherkasy, Cherkasy Regional Hospital, ads, peds, V. Paramonov (60 (60) 22/38)
Kiev, Kiev Regional Hospital, ads, peds, I. Gartovska (27 (27) 0/27)
Kiev, Kiev BMT Center MNPE KBMTC, ads, peds, S.Korenkova, B.Medvediev, O.Apanasenko, V.Boiko, M.Miahkova (52 (56) 21/31)
Kiev, Feofaniya Clinical Hospital, ads, S. Klymenko (25 (25) 0/25)
Kiev, National Pediatric Specialized Hospital, OHMATDYT, ads, peds, CIC 177, O. Lysytsia (64 (64) 46/18)
Kiev, National Cancer Institute, ads, peds, CIC 10832, Y. Stepanishyna, N. Shokun, A. Burtna, S. Pavlyk, K. Bolgarina, H. Klymniuk (70 (70) 0/70)

Lviv, Institute of Blood Pathology and Transfusion Medicine, ads, O. Tsyapka (22 (33) 0/22)

United Arab Emirates: (1 center: 60 (62) 31/29)
Abu Dhabi, Burjeel Medical City, ads, peds, CIC 1063, P. Kaloyannidis (60 (62) 31/29)

United Kingdom: (48 centers: 4298 (4645) 1520/2778)
Aberdeen, The Royal Infirmary, ads, CIC 344, D.J.Culligan (35 (35) 0/35)
Bath, Royal United Hospital, ads, CIC 619, J. Crowe (22 (22) 0/22)
Belfast, Belfast City Hospital Trust, ads, CIC 268, D. Finnegan (78 (88) 13/65)
Birmingham, Queen Elizabeth Hospital, ads, CIC 387, F. Kinsella (251 (269) 83/168)
Birmingham, The Birmingham Children's Hospital, peds, CIC 781, S. Lawson (31 (39) 20/11)
Blackpool, Victoria Hospital, ads, CIC 832, M. Grey, K. Mason (47 (47) 0/47)
Bournemouth, Royal Bournemouth Hospital, ads, CIC 765, D. Allotey, R. Hall, M. Mangles (17 (22) 0/17)
Bristol, Avon and Royal Hospital for Sick Children, ads, peds, CIC 386, C. Besley, O. Mirci-Danicar (122 (134) 85/37)
Cambridge, Addenbrooke's Hospital, ads, peds, CIC 566, C.Crawley,J Craig (186 (186) 86/100)
Cardiff/Swansea, University Hospital of Wales, ads, peds, CIC 303, K.M.O. Wilson, P. Connor, W. Ingram (121 (130) 39/82)
Cheltenham, Cheltenham General Hospital, ads, CIC 398, A. Rye (25 (25) 0/25)
Coventry, University Hospital Coventry & Warwickshire NHS Trust, ads, CIC 322, F. Jones (28 (30) 0/28)
Dudley, Dudley NHS Trust, ads, CIC 405, S. Fernandes (19 (19) 0/19)
Dundee, Ninewells Hospital, ads, CIC 719, G. Marron (15 (15) 0/15)
Edinburgh, The Western General Hospital, ads, CIC 228, V.L. Campbell, P.H.Roddie, N.Storrar, A.Ivanovs, A.J.M.Broom, J.Buxton, I.Koutsavlis, L.Wotherspoon, (50 (56) 0/50)
Exeter, Royal Devon and Exeter Hospital, ads, CIC 571, P. Kerr (25 (28) 0/25)
Glasgow, Beatson, West of Scotland Cancer Centre, ads, CIC 244, A. Clark, D. Irvine, M. Barrett, D. Maxwell (168 (169) 81/87)
Glasgow, Royal Hospital for Children, peds, CIC 707, B. Gibson (24 (25) 17/7)
Leeds, Yorkshire Hospitals NHS Trust, ads, peds, CIC 254, J. Clay, J Ashcroft, R Patmore (201 (225) 83/118)
Leicester, Royal Infirmary Hospital, ads, CIC 713, M. Martin (83 (88) 21/62)
Liverpool, The Clatterbridge Cancer Centre, ads, CIC 501, M. Saif, G. Toth, T. Seddon, M. Wells, A. Milner, L. Laing, L. Jones, P. Hetherington, L. Credidio, (94 (98) 39/55)
Liverpool, Alder Hay, peds, CIC 773, J. Hayden, H. Heartfield (5 (5) 0/5)
London, Hammersmith Hospitals NHS Trust, ads, CIC 205, J. Apperley, E. Kanfer, E. Olavarria, D. Slade (192 (208) 64/128)
London, Royal Marsden Hospital, ads, peds, CIC 218, E. Nicholson (226 (245) 91/135)
London, University College Hospital, ads, peds, CIC 224, B. Carpenter (325 (346) 97/228)
London, Great Ormond Street Hospital, peds, CIC 243, P. Amrolia, K. Rao, R. Chiesa, J. Silva (74 (85) 65/9)
London, The London Clinic, ads, CIC 263, M. Potter (24 (24) 11/13)
London, London Bridge Hospital, ads, CIC 460, M. Kazmi (16 (16) 0/16)
London, St. George's Hospital, ads, CIC 539, M. Koh, M. Klammer (52 (52) 10/42)
London, King's College Hospital, ads, peds, CIC 763, G. Mufti, V. Potter (226 (252) 80/146)
London, St. Bartholomew's and the Royal London NHS Trust, ads, CIC 768, J. Gribben, S. Montoto,J. Cavenagh, S. Agrawal (158 (177) 43/115)
London, St Mary's Hospital, peds, CIC 866, J. de la Fuente, L. Karnik, A. Gassas (27 (29) 27/0)
Manchester, Central Manchester NHS Trust, peds, CIC 521, R. F. Wynn (54 (63) 46/8)
Manchester, The Royal Infirmary, ads, CIC 601, E. Tholouli (161 (165) 77/84)
Manchester, Christie NHS Trust Hospital, ads, peds, CIC 780, A.Bloor (109 (115) 42/67)
Newcastle upon Tyne, Freeman Hospital, ads, peds, CIC 276, M. Collins, M. Slatter (220 (234) 81/139)
Norwich, The Norfolk and Norwich University Hospital, ads, CIC 391, M. Lawes (21 (21) 0/21)
Nottingham, Nottingham City Hospital, ads, CIC 717, J.L. Byrne (131 (156) 40/91)
Oxford, Cancer and Haematology Centre, Churchill Hospital, ads, CIC 255, A. Peniket, R. Danby, G. Collins, J. Kothari, T. Eyre, K. Ramasamy (128 (128) 62/66)
Oxford, John Radcliffe Children's Hospital, peds, CIC 603, E. Blanco, S. Kennedy, A. Drawbridge (6 (11) 0/6)
Plymouth, University Hospitals Plymouth NHS Trust, ads, CIC 823, P. Medd, H. Hunter, D. Lewis and D. Burns (91 (93) 31/60)
Poole, Poole Hospital NHS Foundation Trust, ads, H. Fernandez, S. Pope (8 (9) 0/8)

Sheffield, Teaching Hospitals NHS Trust, ads, CIC 778, J. Snowden (156 (169) 41/115)
Sheffield, Childrens NHS Foundation, peds, CIC 933, K. Patrick (20 (25) 12/8)
Southampton, University Hospital Southampton NHS Foundation Trust, ads, peds, CIC 704, K. Orchard, D. Richardson (173 (213) 33/140)
Stoke-on-Trent, University Hospitals of North Midlands, ads, CIC 394, S.Pillai, K.Karunanithi, A.Moran (31 (31) 0/31)
Swindon, Great Western Hospital, ads, CIC 608, J. S. Griffith (11 (11) 0/11)
Taunton, Taunton and Somerset NHS Foundation Trust, ads, CIC 708, L. Mihailescu (11 (12) 0/11)

*Late report not included in the analysis or manuscript.
